# Analysis of Dynamic Global Transcriptional Atlas Reveals Common Regulatory Networks of Hormones and Photosynthesis Across *Nicotiana* Varieties in Response to Long-Term Drought

**DOI:** 10.3389/fpls.2020.00672

**Published:** 2020-05-27

**Authors:** Jing Wang, Shihua Zhang, Yunpeng Fu, Tiantian He, Xuewen Wang

**Affiliations:** ^1^College of Tobacco Science, Henan Agricultural University, Zhengzhou, China; ^2^Department of Genetics, University of Georgia, Athens, GA, United States; ^3^College of Life Science and Health, Wuhan University of Science and Technology, Wuhan, China

**Keywords:** abiotic stress, gene expression, regulation network, RNA-Seq, Iso-Seq

## Abstract

Land plants evolve drought acclimation. Existing knowledge of gene regulation mainly comes from short-term drought treatment. However, common regulatory mechanism shared by multiple varieties under long-term drought is little explored. Here we investigated changes in physiology, hormones and transcriptomes in leaves of *Nicotiana* varieties K326 and Basma Xanthi with/without drought treatment at time courses spanning 1 month. Analyses of deep RNA-Seq data and further full-length Iso-Seq data revealed an atlas of dynamic changes of transcripts, spliced isoforms, gene expression, associated Gene Ontology, and metabolism pathways. Fewer differentially expressed genes (DEGs) were induced by drought in high tolerance variety than susceptible variety. Comparison among seven hormone signal pathways identified that genes in both abscisic acid and auxin signaling pathways were highly induced although specific genes were depended on the variety. Common hormone regulatory network analysis revealed that genes encoding clade A protein phosphatase 2C gene (*PP2C*) in abscisic acid pathway was the pivotal hub. Expressional regulation in photosynthesis was also common and variety specific. We conclude that long-term drought inducing gene regulatory networks of hormones and photosynthesis are variety dependent, and *PP2C* is the center of the common hormone regulatory network. Thus, this study improves our understanding of gene regulatory network in drought response.

## Introduction

Plants have evolved to adapt to environmental stresses. Drought, or water deficit, is one of the most adverse abiotic stresses and reduces terrestrial plant productivity, thus threatening our food security. Drought triggers many physiological responses, including wilting, closure of stomatal cells, alterations in metabolism, growth arrest, or even death under severe conditions in plants (Shanker et al., 2014). The intensity and duration of drought determine the degree of damage due to increased osmotic stress (Sarto et al., 2017). Drought causes oxidative stress by inducing the accumulation of toxic reactive oxygen species (ROS) and inducing the antioxidant system (Chaves and Oliveira, 2004). CO_2_ assimilation in photosynthesis decreases, impairing sugar biosynthesis under drought (Tang et al., 2017; Zargar et al., 2017). Phytohormones affect gene expression under drought, especially abscisic acid (ABA) and auxin (Shanker et al., 2014; Burgess and Huang, 2016; Mega et al., 2019). Increasing transcriptome profiles have revealed some transcriptional regulations under drought, including hormone signaling genes and transcription factors (Shanker et al., 2014; Burgess and Huang, 2016; Tang et al., 2017). For example, gene *AP2/EREBP* and *NtWRKY69* are regulated in the air-dried leaves of *Nicotiana tabacum* Burley 21 (Rabara et al., 2015). Changes in protein levels were detected under drought, e.g., decreased levels of heat shock protein 70 and rubisco protein (Shanker et al., 2014). Different varieties in the same plant species have different adaptivity to drought stress, which has both genetic and expressional bases. Some existing studies on drought response in different varieties or species are available but most of them are limited to one variety (Su et al., 2013; Chung et al., 2016; Zenda et al., 2019), or between varieties but not on time series (Kang et al., 2011; Ogbaga et al., 2016; Chen et al., 2019; Marchin et al., 2020). Most of the existing studies are focused on early responses (Chung et al., 2016; Zenda et al., 2019). Some studies on long-term drought reveal a typic developmental inhibition and regulations on many genes’ expression, e.g., genes for transcription factor, carbohydrate metabolism and signal transduction, in several species e.g., in oak (Spieß et al., 2012), *Arabidopsis* (Su et al., 2013) and *Populus* (Yan et al., 2012). Many proteins were found to be regulated after 30 days of different water deficit regimes compared with those in well-watered control in casava (Shan et al., 2018). Those knowledges of existing long-term studies were mainly from comparisons between after treatment and before treatment after a long time. The long-term drought effects on genome-wide gene regulation, especially at time courses, in *N. tabacum* are unknown. Hence, our understanding of the intricacies of gene expression and regulation mechanisms under long-term drought is highly required. To increase a power to identify global and conserved regulatory mechanisms, two varieties of *N. tabacum* and long-term drought treatment with well-watered control will be preferred.

The availability of several whole genome sequences of *Nicotiana* and the high-throughput RNA-Seq based transcriptome analysis facilitate the investigation on common expressional regulation in *Nicotiana* and variety specific regulation at a large scale (Sierro et al., 2014; Wang and Bennetzen, 2015; Xu et al., 2017). *N. tabacum* is a model plant, also a crop, for genetic manipulation, and belongs the same genus *Solanum* as tomato and potato. Therefore, the results from the study in *N. tabacum* could be easily transferred to other plants and crops. *N. tabacum* K326 (K326, flue-cured tobacco) and Basma Xanthi (BX, oriental tobacco) are the most cultured *N. tabacum* varieties and have been observed to have different drought tolerance in field. A comparison of gene regulation in the two varieties could further elucidate the molecular mechanism shared by varieties of *N. tabacum*. This strategy has been successfully applied in the study on *Setaria* (Tang et al., 2017).

Here, we investigated the dynamical changes in phenotype, transcriptome, and hormone levels in treated and control leaves of two *N. tabacum* varieties K326 and BX in response to long-term drought stress at five time courses in a green house. We compared the phenotypic changes to check the difference in drought tolerance. We generated deep transcriptome profiles using RNA-Seq with an Illumina platform and full-length transcripts using Iso-Seq with a PacBio platform with the aim to identify differentially expressed genes (DEGs) and their dynamical changes responsible for common and variety-specific responses to drought at different time stages compared with control. We enriched gene ontology (GO) of DEGs and associated pathways of DEGs, with a focus on hormone signaling transduction. Further analyses revealed that changes of six hormones and regulation of DEGs in hormone pathways conferred the different drought tolerance of two varieties; We also found a common correlated gene regulatory network across multiple hormone signaling pathways in response to drought and gene encoding clade A protein phosphatase 2C (PP2C) is the pivotal hub. In addition, the expressional regulation in photosynthesis gene network was also compared in response to drought. We elucidated common expressional regulatory networks of photosynthesis and hormone signaling in response to drought stress, and variety specific expressional regulation for different drought tolerance in *N. tabacum*.

## Materials and Methods

### Drought Treatment

Two *N. tabacum* varieties K326 and BX were used in the study. For each variety, seedlings were germinated and grown in Hoagland nutrient solution for 45 days with a light/dark cycle of 14/10 h at 30/24°C under illumination with 400 W high-pressure metal halide lights and 70% relative humidity according to previous procedures (Zhang et al., 2018). Then, every two seedlings of a variety were transferred into a single pot with composed soil (soil:sand = 4:1) with the addition of ammonium nitrate (120 mg kg^–1^ soil), calcium superphosphate (400 mg kg^–1^ soil), and potassium sulfate (240 mg kg^–1^ soil) and grown for 35 days with a water content of 65–70% in a green house. The 80th day was recorded as the starting day (d0) of treatment and as the shared control for both control group and drought treatment group at the starting stage. On this day, all pots of each variety were randomly divided into two groups. Group I, as the drought treatment, was watered to maintain a relative water content of 45–50% in the soil. Group II, as the control group, was watered normally to maintain a water content of 65–70%. The water content was detected with FieldScout TDR 350 every day (Spectrum, United States). The fourth and fifth leaves of all plants were tagged at d0 day and used for subsequent leaf sampling. Samples of tagged leaves were collected from more than six plants after 0, 2, 5, 15, and 30 days of treatments for each group, and then samples were labeled as according to variety name and treatment type, and days of treatment (Table 1). Sample collections were done between 9 AM and 10 AM at each sampling day. The samples were frozen in liquid nitrogen immediately and then transferred into −80°C until subsequent analysis. For each variety, at least six plants at each collecting time point for each treatment or control were used in each experiment. Three independent replicates were conducted for all controls and treatment experiments.

**TABLE 1:** Information of sample names and days of treatments.

**Content**	**Information**				
Days after germination	80	82	85	95	110
Days with treatment	0	2	5	15	30
Sample ID of *N.* variety K326 under control	K0	CK2d	CK5d	CK15d	CK30d
Sample ID of *N.* variety K326 under drought	K0	DK2d	DK5d	DK15d	DK30d
Sample ID of *N.* variety BX under control	B0	CB2d	CB5d	CB15d	CB30d
Sample ID of *N.* variety BX under drought	B0	DB2d	DB5d	DB15d	DB30d

The abbreviation of K, B, C, D, and #d represent variety K326, variety Basma (BX), control, drought, and the number of days with treatment or control, respectively. For each variety, at least six plants at each collecting time point for each treatment or control were used in each experiment. Three independent experimental replicates were conducted.

### Measurements of the Plant Height, Biomass, and Size of Leaves

The plant height here was the distance from the surface of the soil near to root to the very top of a plant and was measured from more than six plants. The lengths of the fourth and fifth leaves were measured from the far end, meaning near to the stem of plant, of a petiole to the very tip of a leaf blade, while the widths were measured at the widest part of a leaf at perpendicular direction to the measuring of leaf length. More than twelve leaves were measured. Fresh whole plant was weighted for the biomass. Significant differences were analyzed with Duncan’s multiple range tests with *p* < 0.05. For each variety, at least six plants at each collecting time point for each treatment or control were used in each experiment. Three independent experimental replicates were conducted.

### Plant Hormone Analysis

Fresh leaves (0.2–1.0 g tissue) were used for hormone detection. Hormones including zeatin riboside (ZR), indole-3-acetic acid (IAA), GA3, ABA, brassinosteroid (BR), and methyl jasmonic acid (JA-me) were examined via enzyme-linked immunosorbent assay as previously described (Zhao et al., 2006; Wang et al., 2012). The level of each hormone was calculated as ng per gram of fresh leaves. Significant differences were analyzed with Duncan’s multiple range tests with *p* < 0.05. For each variety, at least six plants at each collecting time point for each treatment or control were used in each experiment. Three independent experimental replicates were conducted.

### Iso-Seq Generation and Transcript Analysis

RNAs from all collected samples of each variety were pooled with equal amounts and used to generate full-length transcripts using single molecule sequencing technology. The sequencing library was prepared following the Iso-Seq protocol (P/N100-377-100-05 and P/N100-377-100-04) as described by Pacific Bioscience. Briefly, cDNA was synthesized with the SMARTer PCR cDNA Synthesis Kit and then amplified by using KAPA HiFi PCR Kits. The SMRTbell template prep Kit 1.0 was used to construct the library. The library from each RNA pool was sequenced on the PacBio Sequel platform to generate Iso-Seq reads for each variety following the manufacturer’s recommendation. SMRTLINK software (version v5.0) from PacBio Bioscience was used for Iso-Seq data analysis. First, ROIs were generated using the minimum 1 pass of the insert, a minimum read quality of 0.80, and a minimum read length of 300. Then, FLNC (full-length non-chimeric) reads containing the 5′ and 3′ adapters used in the library preparation and the poly (A) tail were identified using default settings. FLNC reads were then classified with ICE and polished using Quiver with the default HQ set to 0.99 to generate non-redundant transcripts followed by calibration with Lordec (version 0.5.3) with default settings. Transcripts were then mapped to genome reference and then compared the transcription difference with GMAP (built20170424) by using-f samse -n 0.

### RNA-Seq Data Generation, Abundance Analysis, and Annotation

Total RNA was extracted from collected leaf samples from each of the three experiments independently. For each variety, RNA was extracted from pooled leaves of six biological plants at each collecting time point of treatment or control of each green-house experiment. In total, three RNA preps were obtained from three independent green-house experimental replicates for each time point for each variety under each condition. mRNA was isolated from total RNAs using polyT beads and used to construct an RNA-Seq library following previous procedures (Chen et al., 2017). Approximately 6 G bp RNA-Seq reads of paired-end 150 bp were generated from each sample with an Illumina X Ten platform. RNA-Seq reads were preprocessed to remove low-quality reads (Chen et al., 2017) and then mapped to the corresponding genomic assembly of N. *tabacum* K326 or BX (Sierro et al., 2014; Edwards et al., 2017) with TopHat2 (Kim et al., 2013). Then, the transcript abundance was further measured with Cufflinks (version 2.2.1) (Trapnell et al., 2012). DEGs between the treatment and control at the same time point were identified with DESeq2 (version 1.20.0) (Love et al., 2014) from the read count value using the detailed R script as described previously (Chen et al., 2017). The DEGs were defined as at least twofold changes in abundance and false discovery rate (FDR) < 0.01.

The final set of transcripts were merged from RNA-Seq transcripts and the Iso-seq full-length transcripts and then to generate the representative transcripts, which is defined as the longest transcript via tool cd-hit (Li and Godzik, 2006). The gene annotation was not available for the genome of variety BX but the assembly is available. To maximize the identity of gene annotation, the expressed transcripts of BX were then annotated by similarity search against the annotation of K326 genome first. If the annotation is not available, further annotation was conducted against the NR (non-redundant protein sequences at NCBI), UniProtKB (Swiss-Prot and TrEMBL), KOG (euKaryotic Orthologous Groups) and Pfam databases with HMMER3 with an E-value of 1E-5 using the same procedures previously described (Chen et al., 2017; Li et al., 2017). The further annotation results were used again to annotate the transcripts in K326 if which is not annotated in the assembly. Based on annotation ID, Gene Ontology (GO) terms were retrieved from the Gene Ontology database. GO enrichment was conducted with GOseq (Young et al., 2010). The pathway mapping for k number was conducted using KEGG Automatic Annotation Server (KAAS, version 2.1) (Moriya et al., 2007).

### Regulatory Network Analysis

Regulatory network for crosstalk among the hormone pathways was conducted with Pearson and Spearman correlation analysis between the abundance of all DEGs in hormone signaling pathways and all hormones by using functions built in R language (version 3.2.3) (R Core Team, 2015). The significant correlation was identified as coefficient >0.7 and *p* < 0.05 in both correlation analysis methods. The correlation analysis was done for control and drought treatment independently for each variety. The change of correlation was drawn from the comparison between control and drought treatment of each variety. The common correlation in response to drought was constructed by the presence of correlation in both varieties and then was visualized with Cytoscape (version 3.4.0) (Shannon et al., 2003).

## Results

### Phenotypic Responses to Drought of Two *N*. *tabacum* Varieties

**FIGURE 1:**
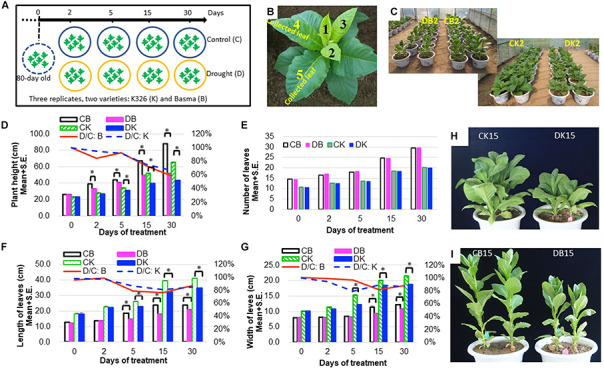
Phenotypic changes of *N. tabacum* varieties K326 and Basma under drought treatment. **(A)** The experimental scheme of treatments and sampling time. **(B)** The fourth and fifth leaves at 80-day old which were tagged and then collected in each time course. **(C)** Plants after 2 days of treatment. **(D)** Changes of the plant height. **(E)** Changes of the total number of leaves. **(F)** Changes of the leaf length. **(G)** Changes of the leaf width. **(H)** K326 plants after 15 days of treatment. **(I)** Basma plants after 15 days of treatment; * for statistical significance at *p* < 0.05. SE, for standard error. CB, DB, CK, DK, D/C:K, and D/C:B represent control B, drought treated B, control K326, drought treated K326, percent of drought to control at the same time point in K326, and percent of drought to control at the same time point in BX, respectively. For all examinations, three experimental replicates with six biological plants in each replicate were conducted.

Eighty-day-old *N. tabacum* plants were treated with drought of 45–50% relative water content or kept under control for 1 month (Figure 1A and Table 1). The drought condition was adopted from our preliminary tests, which allowed plants to survive well until flowering time. Under 45–50% relative water content, *N. tabacum* variety BX successfully develops flower while K326 fails to develop flower after 45 days of drought stress. Therefore, BX has higher drought tolerance than K326. Phenotypic traits of plant were measured, and leaves were sampled after 0 (d0), 2 (d2), 5 (d5), 15 (d15), and 30 (d30) days of treatment (Table 1). A reduced plant height was observed in both K326 and BX varieties as early as d2, which became obvious at d5 (Figures 1C,D,H,I). To avoid a large effect from development, we tagged the fourth and fifth leaves at d0 and focused on these tagged leaves throughout the study (Figure 1B). The leaf length and width were significantly shorter and narrower (*p* < 0.05) under drought condition than under the control condition from d5 in both varieties except that the leaf width of BX had no significant difference compared with that in control at d5 (Figures 1F,G), suggesting a stronger drought tolerance in BX than in K326. However, the total number of leaves was the same for both the drought and control conditions (Figure 1E). The whole plant biomass of both varieties was significantly reduced by the same percent of ∼24% under drought conditions compared with that in the corresponding control at both stages d15 and d30 (Supplementary Figure S1). Together, these phenotypic changes suggest that the drought treatment inhibits vertical growth and leaf expansion, but the tolerance to drought is higher in BX than in K326.

### Transcriptome Data in Leaves Under Control and in Response to Drought

To examine the global changes in gene expression, we used RNA-Seq to profile the transcript abundance in all tagged leaf samples from treatment or control, totaling 27 leaf samples for each variety from five time-courses. More than 20 million 150-bp paired-end reads were generated from each sample, totaling 815 and 795 million reads for K326 and BX, respectively (NCBI accession PRJNA491378), of which 80–86% of reads were mapped to the genome assembly to identify expressed transcripts with TopHat2 (Supplementary Table S1; Kim et al., 2013). In total, we detected 54,439 and 46,526 expressed genes in K326 and BX, respectively.

We also used PacBio Iso-Seq to obtain and analyze full-length transcripts in each variety. We obtained 476,764 and 712,396 full-length non-chimeric reads in K326 and BX, respectively. 68,684 and 43,842 non-redundant transcriptional isoforms were identified in K326 and BX, respectively, after removing redundancy and mapping back to the reference genome. Of these, 93.0 and 99.7% were mapped back to genome assembly. A total of 15.5 and 25.1% were from non-genic regions of the genome annotation in K326 and BX, respectively (Supplementary Table S2). We identified splicing events, including exon skipping in ∼2,500 genes and intron retention in 5,500 genes, by comparing with known gene models.

### Differentially Expressed Genes in Response to Drought at Time Courses

**FIGURE 2:**
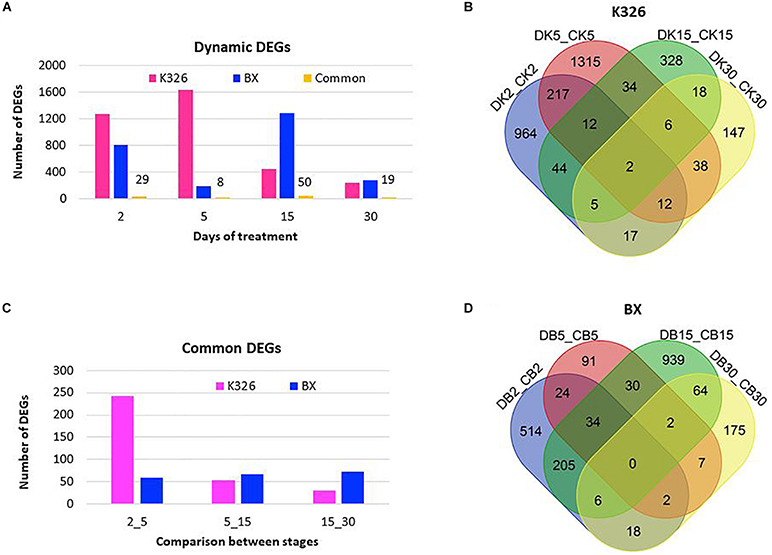
Changes of DEGs in response to drought at time courses in *N. tabacum* varieties K32 and BX. **(A)** Differentially expressed genes (DEGs) with at least twofold changes in transcript abundance (FDR < 0.01) compared with controls at the same stage, and the common DEGs shared by two varieties at each stage, the number above the bar showing the number of shared DEGs. **(B)** Venn diagram showing DEG distribution between time courses in K326. **(C)** Shared DEGs between adjacent treatment stages. **(D)** Venn diagram showing DEG distribution between time courses in BX; Letters D, C, K, and B represent drought treatment, control, K326, and BX, respectively. Numbers 2, 5, 15, and 30 in the sample IDs represent days of drought treatment or control. DEGs were mined from three replicates of RNA-Seq data generated from six biological plants at each stage.

To reveal the expressional regulation, we identified DEGs between the treated samples and control samples at the same time stage, defined as with at least a twofold change of abundance and FDR value <0.01, with DESeq2 (Love et al., 2014). In K326, more DEGs were identified after 2–5 days of drought treatment than after prolonged days (5–30) of drought treatment, indicating a major transcriptional regulation in the early days (Figure 2A). However, only two common DEGs were present across all stages (Figure 2B), indicating expressional regulation was time dependent. Fewer DEGs were identified in BX than in K326. Most DEGs were detected after 15 days of drought treatment in BX, meaning a later peak time of expressional regulation in BX than K326 (Figure 2A), and no common DEG was presented across all stages in BX (Figure 2D). Common regulated genes which shared similar sequence of transcripts between two varieties were compared, and 29, 8, 50 and 19 common DEGs were identified at d2, d5, d15, and d30, indicating common regulation to drought between varieties (Figure 2A). Comparisons of common DEGs between adjacent stages revealed more common DEGs between early time points of d2 and d5 than other time points in K326, while the number of common DEGs were relatively stable across time points in BX (Figure 2C). By combining phenotypic changes with DEG changes, we found that a high drought tolerance was associated with less change of gene expression in early drought-treatment stages.

### GO Functions and Metabolism Pathway Networks Involved by DEGs

To further understand the function of DEGs, we conducted GO enrichment analysis (*p* < 0.05) with GOseq (Young et al., 2010) and compared the GO terms between stages. In total, up to 250 GO terms were enriched. In both varieties, most GO terms of DEGs were over-represented instead of under-represented (Supplementary Figure S2 and Supplementary Table S3). More enriched GO terms were found at early stages than at late stages in k326, except that stage d5 in BX had fewer enriched GO terms than other stages (Supplementary Figure S2A). In the cellular component category, the most affected GO terms were associated with photosynthesis and chloroplast (Supplementary Figure S2B). In the molecular function category, major enriched GO terms included many enzyme activities, transporters, and ATP binding (Supplementary Figure S2C). In the biological process category, enriched GO terms were oxidation-reduction process, response to water deprivation, and response to stress (Supplementary Figure S2D). The photosynthesis process and hormone signal pathways were also enriched (Supplementary Tables S4, S5). In addition, some GO terms were enriched only in one variety or in specific stages (Supplementary Figure S2).

We further investigated the metabolism pathway networks enriched by DEGs against the KEGG database (Supplementary Figures S3A–D). In K326, photosynthesis was significantly affected at d2. Other significant pathways were involved, including protein processing in the endoplasmic reticulum and galactose metabolism for hemicellulose at d5. At d15, the pathways including flavonoid biosynthesis, porphyrin and chlorophyll metabolism were enriched. At d30, the pathways of arginine and proline metabolism, nitrogen metabolism, glyoxylate and dicarboxylate metabolism, and selenocompound metabolism were enriched (*q* < 0.05). Compared with K326, BX exhibited a different pattern of enriched pathways (Supplementary Figures S3E–H). At d2, the significantly enriched pathways (*q* < 0.05) were plant hormone signal transduction, starch and sucrose metabolism, and pentose and glucoronate interconversions. At d5, carotenoid biosynthesis and plant hormone signal transduction were enriched. At d15, plant hormone signal transduction, starch and sucrose metabolism and phenylpropanoid biosynthesis were enriched (*q* < 0.05). At d30, glycerolipid metabolism, vitamin B6 metabolism, protein processing in the endoplasmic reticulum, and amino sugar and nucleotide sugar metabolism were enriched. Similar to the GO enrichment result, fewer DEGs were enriched in pathways in BX than in K326. Other enriched pathways, e.g., lipid-associated pathways, were common between varieties but presented at different stages.

### Expressional Regulation in Photosynthesis Network in Response to Drought Stress

We identified 56 and 11 DEGs that were annotated to encode 23 and 8 proteins in the enriched photosynthesis process in K326 and BX, respectively, suggesting greater effects on gene expression in K326. Of those, seven encoded common proteins were identified between two varieties, suggesting common expressional regulation of photosynthesis network. These common proteins were known as plastocyanin, ferredoxin, photosystem I subunit VI, photosystem II CP43 chlorophyll apoprotein, light-harvesting complex I chlorophyll a/b binding protein 3, light-harvesting complex II chlorophyll a/b binding protein 4, and photosystem I subunit PsaO (Supplementary Table S6). We also identified variety-specific proteins encoded by DEGs, e.g., protein F-type H+-transporting ATPase subunit alpha in K326. An overall trend of expressional regulation was that drought treatment reduced the transcript levels of most DEGs in the photosynthesis network (Supplementary Figure S4). Interestingly, the abundances of several DEGs were increased. In K326, genes *0001791g0030* and *0000134g0030* encoding a photosystem II 10 kDa protein, genes *0009833g0010* and *0013140g0020* encoding ferredoxin, and gene *N_11965* encoding photosystem II CP43 chlorophyll apoprotein showed increased expressional pattern at early treatment stages. In BX, gene *N_34854* encoding ferredoxin and gene *N_32233* encoding photosystem II CP43 chlorophyll apoprotein showed increased expressional pattern at stages d2 through d15 (Supplementary Figure S4 and Supplementary Table S6).

### Expressional Regulation in Hormone Signal Transduction Network in Response to Drought

**FIGURE 3:**
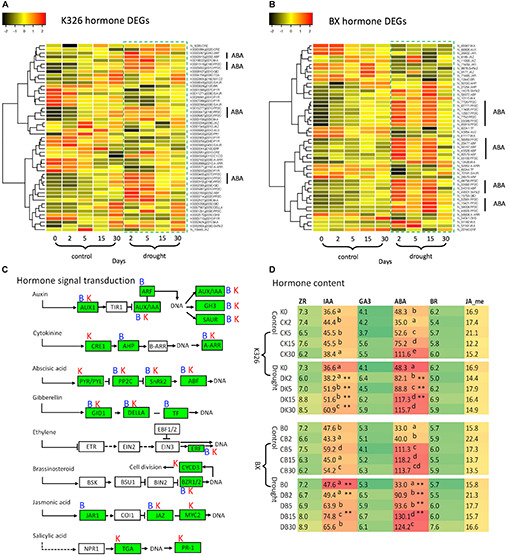
Comparison of hormones and DEGs in hormone signaling pathways under drought stress. **(A)** Patterns of DEG expression level after log_2_(FPKM + 1) transformation and *Z*-score scaling in variety *N. tabacum* K326. **(B)** Patterns of DEG expression level after log_2_(FPKM + 1) transformation and *Z*-score scaling in variety *N. tabacum* BX. **(C)** Hormone signal transduction pathways, which were drawn based on the information of module ko04075 in the KEGG database (https://www.genome.jp/kegg, accessed on November 18, 2019); Letters “B” and “K” represent the DEG presence at BX and K326, respectively. **(D)** Abundances of six hormones. The hormone content was calculated as ng per gram of fresh leaves. Up script letters denote the significant differences from Duncan’s multiple range tests with *p* < 0.05. Asterisk ** represents significance at *p* < 0.05 and *p* < 0.01 between drought and control at the same time stage. AUX1, auxin influx carrier; AUX1/IAA, auxin-responsive protein IAA; ARF, auxin response factor; GH3, auxin responsive GH3 gene family; SAUR, SAUR family protein; CRE1, *Arabidopsis* histidine kinase 2/3/4; AHP, histidine-containing phosphotransferase; A-ARR, two-component response regulator ARR-A family; PYR/PYL, abscisic acid receptor PYR/PYL family; PP2C, clade A protein phosphatase 2C; SnRk2, serine/threonine-protein kinase domain SRK2 subgroup 3; ABF, ABA-responsive element binding factor; GID1, gibberellin receptor GID1; DELLA, DELLA protein; TF, phytochrome-interacting factor 4; ERF, ethylene-responsive transcription factor 1; BZR1/2, brassinosteroid resistant 1/2; CYCD3, cyclin D3; JAR1, jasmonic acid-amino synthetase; JAZ, jasmonate ZIM domain-containing protein; TGA, transcription factor TGA; PR-1, pathogenesis-related protein 1; ZR, zeatin riboside as a kind of cytokinin; IAA, indole-3-acetic acid; ABA, abscisic acid; BR, brassinosteroids; JA-me, methyl jasmonic acid. Expression data and hormone levels were obtained from three replicates, each with six biological plants at each stage.

Since hormone signal transduction pathways were enriched by DEGs, we further investigated the regulation of these DEGs. 51 and 50 DEGs were identified in varieties K326 and BX, respectively, which were involved with seven hormones including ABA, auxin, cytokinin, gibberellin, brassinosteroid, jasmonic acid, and ethylene (Figure 3). We found these DEGs were annotated to encode 19 and 16 proteins in K326 and BX, respectively, most of which have been known as transcription factors (Supplementary Table S7). However, in the phytohormone salicylic acid (SA) pathway, no DEG was identified in BX while two DEGs encoding the TGA and PR1 proteins were regulated in K326 (Figure 3A), which indicated that regulation of gene expression in the SA signal was variety specific.

More DEGs were enriched in both ABA and IAA signal pathways than in other hormone pathways (Figures 3A,B). In the ABA signaling pathway, we found multiple DEGs encoding phosphatase protein PP2C and transcription factor ABF in both varieties. Abundance of most *PP2C* and *ABF* transcripts was upregulated in response to drought compared with that in control at the same time point (Figures 3A,B). Multiple genes were also identified to encode an ABA receptor called PYR; However, the transcript levels of seven *PYRs* were down-regulated in K326 but unchanged in BX throughout drought treatment (Figures 3A,B).

Multiple genes encoding IAA proteins were identified in both varieties. The transcript levels of four *IAA* genes were upregulated in both varieties in response to drought at early stages, while two additional *IAA* genes in BX were down-regulated in response to drought (Figures 3A,B).

The changes of those DEGs led us to hypothesize that levels of hormone IAA and ABA were regulated in response to drought. To test this, further measurement of hormones showed that IAA and ABA were the most abundant hormones, at least two times higher than the levels of other hormones in both varieties. IAA levels were slightly increased in control conditions (Figure 3D) which could be needed for the observed growth of leaf during the whole experimental period (Figures 1F,G). IAA levels were highly increased at stages d5, d15, and d30 in response to drought compared with that in control in both varieties. The ABA levels were significantly increased between stage d2 and d15 (*p* < 0.05) in both varieties, except at stage d5 in BX, in response to drought compared with that in control (Figure 3D).

### Crosstalking Network Among Multiple Hormone Signaling Pathways

Hormone signaling pathways cross-talk, e.g., between ABA and IAA signaling pathways (Park et al., 2009). To understand the crosstalk between DEGs in multiple hormone pathways, we further analyzed the correlation network (coefficient >0.7 and *p* < 0.05) between abundance of DEGs, and between hormone levels and abundance of DEGs. Results revealed expressional networks among seven examined hormone signaling pathways, which had difference between two varieties (Supplementary Figure S5). In the variety K326, when treated with drought, the major change in crosstalk networks was found between ABA signaling pathway and auxin signaling pathway, and between ABA signaling pathway and salicylic acid pathway. Even within the ABA signaling pathway, most *PP2C* genes were found to be negatively co-expressed with *PYR* genes under drought while it was not under control. It is known that the *PYR* gene inhibits *PP2C* gene expression (Park et al., 2009), so the inhibition was significantly enhanced in response to drought in K326 (Supplementary Figures S5A,B).

In variety BX, negative correlations between auxin signaling genes and *PP2C* in ABA signaling pathway were observed under control while they were absent with drought treatment. Positive correlations of between expression of *PP2C* genes and *GID*, hormone GA3’s level in GA signal pathway as well as three negative correlations with other hormone signal genes were observed under drought treatment while no correlation was found under control. Even within ABA signaling pathway, more *ABF* genes were positively co-expressed with genes *PP2C* with drought treatment than under control (Supplementary Figures S5C,D).

**FIGURE 4:**
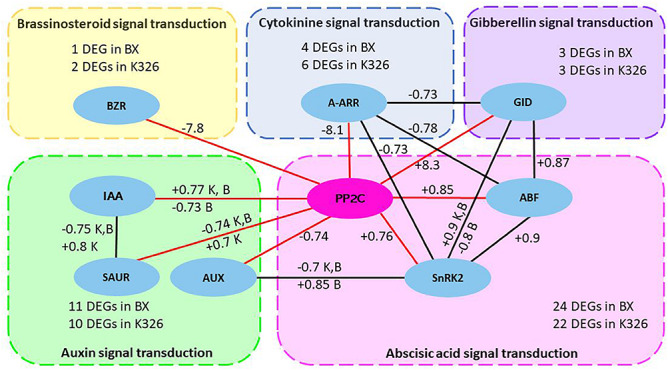
Common regulatory network of hormone signaling pathways in response to drought between two *Nicotiana* varieties.

A common network was further investigated by checking the shared crosstalk of hormone signaling pathways in both varieties. The common significant correlations (coefficient >0.7 and *p* < 0.05) were examined in any pair of hormones or DEGs including 51 and 50 DEGs from variety K326 and BX, respectively (Supplementary Table S8). A common crosstalk network among signaling pathways of five hormones IAA, ABA, GA, BR, and cytokinine in response to drought was constructed by using Cytoscape (version 3.4.0) (Figure 4). In this network, most correlations were shared by the same type of positive and negative regulation between K326 and BX. Positive correlations were identified between *PP2C* and DEGs *ABF* and ABA dependent serine/threonine-protein kinase domain SRK2 subgroup 3 (*SnRK2*) in abscisic acid signal pathway, or *GID* in gibberellin signal pathway. Negative correlations were identified between *PP2C* and DEG *A-ARR* in cytokinine signal pathway, or DEG *BZR* in brassinosteroid signal pathway, which indicating an inhibition role in growth. The correlation between *PP2C* and DEGs in auxin signal pathway is negative and/or positive. Surprisingly, four additional regulations are also found in K326 or BX besides common correlated regulations. For example, a common positively correlated regulation between genes *PP2C* and *IAA* is presented in both varieties and an additional negatively correlated regulation between these two DEGs is also identified in variety BX. The ABA signaling pathway is the most correlated to other hormone signal pathways. Thus, gene *PP2C* in the ABA signaling pathway was the pivotal hub of the common network, which interacted with more DEGs than any other DEGs in hormone pathways (Figure 4).

The common crosstalk network between five hormone signaling pathways was constructed by correlation coefficient >0.7 and *p* < 0.05 from Pearson analysis and was shared by *Nicotiana* varieties K326 and BX. The node in eclipse represents differentially expressed gene (DEG) in hormone signaling pathways and the DEG list for each hormone signaling pathway is available from Supplementary File and Supplementary Table S8. The edge shows the correlation. Each box with dash line represents a hormone signaling pathway. Red edges show the pivotal correlation of gene *PP2C* with other DEGs. The number next to edge represents the mean of positive (+) or negative (−) coefficient of correlated regulation. The letter K or B will be added if the correlation has both positive and negative relationship which come from multiple copies of DEGs encoding the same type of protein. K, variety K326; B variety BX; BZR1/2, brassinosteroid resistant 1/2; IAA, auxin-responsive protein IAA; SAUR, SAUR family protein gene; AUX1, auxin influx carrier gene; A-ARR, two-component response regulator ARR-A family gene; PP2C, clade A protein phosphatase 2C gene; SnRk2, serine/threonine-protein kinase domain SRK2 subgroup 3 gene; ABF, ABA-responsive element binding factor gene; GID, gibberellin receptor GID gene.

## Discussion

Studies on responses to drought have been reported in many plant species at phenotypic, physiological, and biochemical, genetic, and gene expressional levels. However, most of them have focused on early or long-time responses, or in a single variety with or without time series, or between multiple varieties (often without time series), or on media, or non-industrial crops, which limit the application in field improvement in real farming. Different durations of drought treatment were used in existing reports (Kang et al., 2011; Spieß et al., 2012; Yan et al., 2012; Chung et al., 2016; Ogbaga et al., 2016; Shan et al., 2018; Chen et al., 2019; Zenda et al., 2019; Marchin et al., 2020). We propose that long-term drought stress should be relative long time e.g., many weeks, usually spanning one or more developmental stages, which may cause severe damage to growth and yield during farming. Short-term drought stress is only several hours and very few days, which induces early responses and may not cause severe damage. Here our time-course analyses examined both short-term and long-term drought treatments to two *Nicotiana* varieties with real-time control in green house in farming practice. Our results of global, deep and full-length transcriptomic atlas revealed dynamic expressional regulations across varieties induced by drought. The results of common expressional regulation and variety specific regulation have dissected the molecular basis of shared responses and specific tolerances evolved in varieties. We conclude that fewer DEGs are associated with higher drought tolerance in *Nicotiana*. Expressional regulation of DEGs in plant hormone signaling pathways and photosynthesis pathways is one of key gene regulation in response to long-term drought, especially the pivotal *PP2C* regulation in the crosslinked hormone network. Previously, *PP2C* was also reported to be involved in defense after a long-term drought stress across a full growth season in oak tree (Spieß et al., 2012). Hormone ABA inhibits PP2C via the PYR (Park et al., 2009). The finding of increased IAA should expect to see lowly expressed *PP2C*. However, increased levels of most *PP2Cs* were observed which may indicate a functional diversity of multiple *PP2C* genes and *PYR* genes in leaves. In addition, this complexity of *PP2Cs* positively or negatively correlated with many other DEGs in the common regulation network identified here together may explain the distinct levels of *PP2Cs* and the hub role in the regulation network. The detailed function of each *PP2C* gene is worthy of further characterization in near future by using overexpression and knock-out analysis as previously described (Zhou et al., 2020). The *PP2C* regulation should be applicable to other varieties of *N. tabacum* or other crops in *Solanaceae* family since it is obtained from common hormone regulatory network across time courses and varieties. Thus, we obtained new insights for expressional regulation in long-term drought stress, which improves our understanding of regulatory network in response to stress.

Hormones regulate a series of responses under drought (Shanker et al., 2014). Drought acclimation, which is known to modulate a higher tolerance, at least through ABA and cytokinin (Li et al., 2016). Previous microarray-based expression analyses showed that both ABA and JA were regulated during the long-term stress through seed development in *Arabidopsis* (Su et al., 2013). Many genes involved in hormone pathways were also significantly regulated after long-term stress under different water deficit regimes (Yan et al., 2012). Here the long-term drought induced regulation of DEGs in the ABA, cytokinin and IAA signaling pathways in leaves; thus, associated DEGs with those hormones should also be responsible for drought acclimation. Result from previous report shows that IAA could antagonize the increased ABA level via phosphate-related degradation through MAPK signaling (Park et al., 2009). Therefore, the observed increase of IAA level in leaves in our study could play role to eliminate the increased ABA level during long-term drought stress, thus, to reduce the drought inhibition on growth as the result of drought acclimation. The similar interaction of ABA with IAA during drought was also identified in other plant like *Populus* (Zhou et al., 2020). But another study in roots showed that ABA accumulation promotes auxin transport in the root tip to enhance the proton efflux and ATPase activity to maintain root growth under drought (Xu et al., 2013). Therefore, the increase of IAA level in leaves in our study might be, in return, resulted from increased ABA level. Here, both hormones IAA and ABA were also increased when the plant grew in control groups. The increase of IAA could be the need of the continued growing of leaf (Figures 1E,F). In the other hand, when leaf grows bigger and bigger, the leaf is aging which could also increases the ABA levels. In field farming practice, the leaf growth will stop earlier in BX than in K326, and at the end of d30 treatment experiment, the leaf of BX is very close to mature stage. This phenomenon well matches an early increase of ABA in BX than in K326 even in control groups. In addition, TFs identified here in hormone signal transduction are known to regulate trichome exudates on the leaf and leaf senescence in response to stress (Lim et al., 2007; Jibran et al., 2013), which could help to reduce water loss via transpiration and shorten life cycle of a plant as an acclimation. To summarize, these associated DEGs identified in this study could be the key regulatory genes for drought acclimation, which is of high priority in further studies.

Photosynthesis was reduced in plants in response to many stresses, such as cold, heat, drought, light quality, and day length (Wang et al., 2011, 2019; Shanker et al., 2014; Li et al., 2019), which suggests a common regulation under stresses. Here, the photosynthesis was reduced in both varieties. The finding of regulation on both hormones and photosynthesis in our results could be interpreted as integrated response to drought stress via linked regulation. For instance, the DEG called *SnRK1* in ABA signaling could sense energy depletion (Hulsmans et al., 2017), which was supported by reduced ATP metabolism in our GO enrichment result.

Many plant species regulate stomatal closure and transpiration in response to short-term drought (Zhu, 2016). The maintenance of plant growth under long-term drought depends crucially on soil water uptake by root system (Brunner et al., 2015). Several reports show that moderate long-term drought promotes root growth to increase drought resistance (Jeong et al., 2013; Lee et al., 2016; Yu et al., 2016). For the leafy crops, like tobacco, the gene regulation in leaf is more important than that in roots once plant can survive under moderate long-term drought. The gene regulation in leaves under short-term and long-term drought is not well understood. Here, we applied time-course based investigation, so gene regulation under both short-term and long-term drought was explored. The results of DEGs’ expression patterns and enrichment of associated GOs and pathways across time courses provide a detailed dynamic expressional regulatory atlas in response to drought stress. We conclude that a typic regulatory pattern is that the variety with high tolerance to drought has fewer DEGs at the early stages of long-term drought stress, while the variety with low tolerance to drought has more genes affected by drought at early stage in *N. tabacum*, which was supported by photosynthesis and hormone signaling in K326 and BX. This may be associated with high susceptibility in low drought tolerant variety. Previously studies reported regulation in response to drought including ROS, reduced photosynthesis, ABA, and transcription factor (Shanker et al., 2014; Chaves and Oliveira, 2004; Mega et al., 2019). Here we found consistent regulation on some common changes including photosynthesis, hormone signal transduction, energy, oxidation-reduction, response to water deprivation and stress. We also found some new aspects in DEGs and their GOs between varieties. The differences in GO dynamics between the high-tolerance and low-tolerance varieties reflect the variety specific regulation. Thus, the associated DEGs provide good potential candidates for further investigation on gene function, which confer difference in drought tolerance. In addition, some DEGs were variety dependent, meaning that we should pay attention to the limitation when we try to infer the regulation from one variety to another variety even within a species, which is not addressed in most existing studies on only one variety.

## Data Availability Statement

The RNA-Seq data and Iso-Seq data used in this study are freely available at NCBI short sequence archive under project accession PRJNA491378.

## Author Contributions

YF and XW: conceptualization. JW, YF, and XW: methodology. XW: software. JW, TH, and SZ: formal analysis. JW, SZ, YF, TH, and XW: investigation. JW and YF: resources. JW and XW: writing-original draft preparation. JW, YF, and XW: writing-review and editing.

## Conflict of Interest

The authors declare that the research was conducted in the absence of any commercial or financial relationships that could be construed as a potential conflict of interest.
