# Supplementary material for: Analysis of Dynamic Global Transcriptional Atlas Reveals Common Regulatory Networks of Hormones and Photosynthesis Across Nicotiana Varieties in Response to Long-Term Drought
Source: Front Plant Sci. 2020 May 27;11:672. doi: 10.3389/fpls.2020.00672 (PMC7266868; doi:10.3389/fpls.2020.00672)
Supplement: Supplementary file 1 [file Data_Sheet_1.PDF]

Analysis of Dynamic Global Transcriptional Atlas Reveals Common Regulatory Networks of Hormones and Photosynthesis across *Nicotiana* Varieties in Response to Long-term Drought

Jing Wang [1], Shihua Zhang [2,3], Yunpeng Fu [1,*], Tiantian He [1], Xuewen Wang [2,*]

Supplementary information

Tables:

Table S1 Summary of RNA-Seq profiles from Illumina sequencing 2
Table S2 Summary of single-molecule transcriptome profiles from PacBio Iso-Seq 4
Table S3 Comparisons of GO enrichment for DEGs between varieties and stages 6
Table S4 Enriched GO terms of DEGs and comparison across stages in variety K326 11
Table S5 Enriched GO terms of DEGs and comparison across stages in variety BX 11
Table S6 Comparison of DEGs in photosynthesis in Nicotiana varieties under drought 11
Table S7 Comparison of the DEGs encoding proteins in hormone signal transduction in response to drought stress in Nicotiana varieties 13
Table S8 DEGs and involved pathway node in the correlation network............................... 15

Figures:

Figure S1. Biomass changes of whole plant of two N. varieties under drought..................... 17
Figure S2. Comparison of top enriched GO terms of DEGs in response to drought 20
Figure S3. Comparison of enriched metabolism pathways involved by DEGs under drought 22
Figure S4 Comparison of expression patterns of DEGs in photosynthesis under drought .. 24
Figure S5 Correlation network of hormones and DEGs in hormones signaling pathways .. 27

Table S1 Summary of RNA-Seq profiles from Illumina sequencing

Sample	Species	Treatment(d)	Replicates	Q30(%)	Total Reads	Mapped Reads	Uniq Mapped Reads
B0-1	Basma	control0	1	91.06	43,790,812	36,977,952 (84.44%)	35,481,761 (81.03%)
B0-2	Basma	control0	2	90.22	48,212,626	40,066,603 (83.10%)	38,374,923 (79.60%)
B0-3	Basma	control0	3	91.54	45,637,472	38,309,170 (83.94%)	36,742,993 (80.51%)
CB2-1	Basma	control2	1	90.61	48,651,104	40,916,612 (84.10%)	39,251,348 (80.68%)
CB2-2	Basma	control2	2	91.82	54,115,956	45,600,786 (84.26%)	43,616,516 (80.60%)
CB2-3	Basma	control2	3	90.22	44,975,166	36,952,453 (82.16%)	35,368,726 (78.64%)
DB2-1	Basma	drought2	1	90.74	47,340,016	39,586,329 (83.62%)	37,801,808 (79.85%)
DB2-2	Basma	drought2	2	90.99	48,063,178	39,905,857 (83.03%)	38,093,585 (79.26%)
DB2-3	Basma	drought2	3	90.76	49,803,300	41,407,209 (83.14%)	39,600,516 (79.51%)
CB5-1	Basma	control5	1	90.73	57,664,658	48,116,769 (83.44%)	45,950,119 (79.69%)
CB5-2	Basma	control5	2	91.21	42,690,424	35,993,766 (84.31%)	34,414,446 (80.61%)
CB5-3	Basma	control5	3	88.93	41,868,378	34,023,479 (81.26%)	29,776,420 (71.12%)
DB5-1	Basma	drought5	1	90.72	40,730,004	33,873,790 (83.17%)	32,239,983 (79.16%)
DB5-2	Basma	drought5	2	89.97	47,163,056	38,845,340 (82.36%)	37,094,696 (78.65%)
DB5-3	Basma	drought5	3	91.02	46,832,260	39,205,557 (83.71%)	37,257,357 (79.55%)
CB15-1	Basma	control15	1	89.88	44,525,480	36,511,251 (82.00%)	32,978,691 (74.07%)
CB15-2	Basma	control15	2	91.1	42,122,112	34,995,063 (83.08%)	33,364,862 (79.21%)
CB15-3	Basma	control15	3	90.97	53,345,204	44,048,369 (82.57%)	42,020,390 (78.77%)
DB15-1	Basma	drought15	1	90.68	50,850,358	41,982,968 (82.56%)	40,195,526 (79.05%)
DB15-2	Basma	drought15	2	91.16	49,732,530	41,411,081 (83.27%)	39,534,839 (79.49%)
DB15-3	Basma	drought15	3	90.41	47,188,664	38,449,049 (81.48%)	36,502,152 (77.35%)
CB30-1	Basma	control30	1	90.09	51,730,450	42,669,843 (82.48%)	40,756,888 (78.79%)
CB30-2	Basma	control30	2	89.31	49,463,962	40,828,190 (82.54%)	39,028,050 (78.90%)
CB30-3	Basma	control30	3	89.58	50,729,022	41,546,510 (81.90%)	39,705,607 (78.27%)
DB30-1	Basma	drought30	1	89.39	47,567,960	38,805,667 (81.58%)	36,462,883 (76.65%)
DB30-2	Basma	drought30	2	89.02	48,878,448	39,787,720 (81.40%)	37,772,455 (77.28%)

DB30-3	Basma	drought30	3	89.63	46,133,230	37,988,920 (82.35%)	36,029,748 (78.10%)
total					1,589,032,090		
K0-1	k326	control0	1	93.01	41,523,862	35,920,106 (86.50%)	34,405,625 (82.86%)
K0-2	k326	control0	2	92.92	40,927,306	35,532,313 (86.82%)	34,067,962 (83.24%)
K0-3	k326	control0	3	88.52	40,660,712	33,189,584 (81.63%)	31,834,630 (78.29%)
CK2-1	k326	control2	1	89.88	53,565,732	44,989,126 (83.99%)	43,161,515 (80.58%)
CK2-2	k326	control2	2	90.38	50,839,764	42,735,261 (84.06%)	40,988,794 (80.62%)
CK2-3	k326	control2	3	89.05	57,169,820	47,327,765 (82.78%)	45,291,391 (79.22%)
DK2-1	k326	drought2	1	89.87	52,648,784	44,044,112 (83.66%)	42,189,968 (80.13%)
DK2-2	k326	drought2	2	90.05	53,427,044	45,354,660 (84.89%)	43,040,037 (80.56%)
DK2-3	k326	drought2	3	88.49	46,802,712	38,733,506 (82.76%)	37,088,627 (79.24%)
CK5-1	k326	control5	1	91.01	41,281,116	35,256,343 (85.41%)	33,734,733 (81.72%)
CK5-2	k326	control5	2	90.53	44,192,368	36,929,643 (83.57%)	35,336,936 (79.96%)
CK5-3	k326	control5	3	90.94	47,278,018	39,874,158 (84.34%)	38,257,613 (80.92%)
DK5-1	k326	drought5	1	92.38	49,107,420	42,171,722 (85.88%)	40,290,664 (82.05%)
DK5-2	k326	drought5	2	89.93	73,153,936	61,062,230 (83.47%)	58,241,977 (79.62%)
DK5-3	k326	drought5	3	89.16	44,480,200	36,604,008 (82.29%)	34,754,023 (78.13%)
CK15-1	k326	control15	1	91.22	50,944,568	42,858,081 (84.13%)	40,424,079 (79.35%)
CK15-2	k326	control15	2	90.92	44,841,194	37,564,127 (83.77%)	35,851,909 (79.95%)
CK15-3	k326	control15	3	90.95	55,136,098	46,119,314 (83.65%)	44,122,342 (80.02%)
DK15-1	k326	drought15	1	90.17	60,518,700	50,291,857 (83.10%)	47,565,431 (78.60%)
DK15-2	k326	drought15	2	90.25	47,730,902	39,684,948 (83.14%)	38,044,088 (79.71%)
DK15-3	k326	drought15	3	90.59	48,320,528	40,303,828 (83.41%)	38,612,879 (79.91%)
CK30-1	k326	control30	1	89.9	47,664,132	39,619,803 (83.12%)	37,407,033 (78.48%)
CK30-2	k326	control30	2	90.07	50,221,030	41,923,955 (83.48%)	39,982,961 (79.61%)
CK30-3	k326	control30	3	91.4	47,318,604	40,324,087 (85.22%)	38,361,632 (81.07%)
DK30-1	k326	drought30	1	89.37	47,280,022	39,274,751 (83.07%)	37,513,322 (79.34%)

DK30-2	k326	drought30	2	90.07	54,132,982	45,279,996 (83.65%)	43,064,801 (79.55%)
DK30-3	k326	drought30	3	88.76	47,440,864	38,927,496 (82.05%)	36,819,361 (77.61%)
total					1,630,365,336		

Table S2 Summary of single-molecule transcriptome profiles from PacBio Iso-Seq

Category	Sub-category	K326	Percent	Basma	Percent
**Reads of insert**	Total number	**570,916**		**902,600**	
	Mean passes per insert	22		13	
**Full-length reads**	Full-length reads	**499,132**	**87.4%**	**744,700**	**82.5%**
	Full-length non-chimeric reads	476,764	83.5%	712,396	78.9%
	Full-length non-chimeric bases (bp)	1,080,260,739		1,599,702,183	
	Mean length (bp)	2,238		2,226	
**Consensus sequence**	Total number	**224,468**	**45.0%**	**350,065**	**47.0%**
	Mean length of consensus sequence (bp)	2,324		2,257	
	Bases after Illumina RNA-Seq correction (bp)	522,261,085		790,407,465	
	Number of high base-quality (>0.99) sequence	43,137		59,551	
	Bases of high base-quality (>0.99) sequence	94,566,342		129,418,810	
	Fusion transcripts	698		5,342	
**Isoform mapping**	Total number of isoforms	**43,842**		**68,684**	
	Number of isoforms mapped to known gene	37,043	84.5%	51,418	74.9%

	Number of isoforms mapped to no gene region	6,799	15.5%	17,266	25.1%
	Total mapped known genes	13,975		19,103	
	Total predicted genes in genome	35,519		72,819	
	Total mapped rate	93.0%		99.7%	
	Mean consensus number per isoform	2.48		3.38	
**Splicing**					
	Exon skipping / genes	3,219	/2,164	2,991	/2,035
	Alternative donor site / genes	4,390	/2,772	5,119	/2,288
	Alternative acceptor site / genes	4,315	/2,591	5,264	/3,104
	Intron retention / genes	18,127	/5,417	23,168	/6,844
	Alternative position / genes	4,073	/2,235	6,464	/3,157
	Other	5,082	/2,279	6,007	/2,718

Table S3 Comparisons of GO enrichment for DEGs between varieties and stages

GO_term	Variety_Stage	pvalue	Gene_count	category
oxidation-reduction process	B2	0.000502	52	BP
regulation of transcription, DNA-templated	B2	2.54E-04	31	BP
carbohydrate metabolic process	B2	8.83E-04	16	BP
response to water deprivation	B2	2.01E-07	15	BP
cellular glucan metabolic process	B2	1.22E-13	14	BP
lipid metabolic process	B2	0.000208	11	BP
response to stress	B2	0.046034	8	BP
protein dephosphorylation	B2	0.000802	8	BP
negative regulation of catalytic activity	B2	0.003114	6	BP
cell wall modification	B2	0.001829	5	BP
oxidation-reduction process	K2	8.46E-07	108	BP
translation	K2	1.76E-03	30	BP
isopentenyl diphosphate biosynthetic process	K2	1.00E-11	29	BP
photosynthesis, light harvesting	K2	3.13E-28	29	BP
rRNA processing	K2	9.91E-11	27	BP
protein-chromophore linkage	K2	4.16E-18	25	BP
photosynthesis	K2	1.81E-12	25	BP
chlorophyll biosynthetic process	K2	4.29E-12	22	BP
cysteine biosynthetic process	K2	1.33E-09	21	BP
pentose-phosphate shunt	K2	2.66E-07	20	BP
regulation of transcription, DNA-templated	B5	0.030717	8	BP
response to water deprivation	B5	4.78E-06	7	BP
response to cadmium ion	B5	0.017191	5	BP
response to stress	B5	2.81E-03	5	BP
starch biosynthetic process	B5	0.018325	4	BP
response to auxin	B5	1.48E-02	3	BP
protein dephosphorylation	B5	0.010505	3	BP
transcription, DNA-templated	B5	0.042558	3	BP
regulation of cellular macromolecule biosynthetic process	B5	5.51E-04	2	BP
regulation of primary metabolic process	B5	0.013734	2	BP
oxidation-reduction process	K5	5.76E-06	125	BP
response to stress	K5	2.19E-23	50	BP
protein folding	K5	6.66E-19	50	BP
carbohydrate metabolic process	K5	0.00748	28	BP
response to heat	K5	2.49E-10	26	BP
response to high light intensity	K5	5.55E-12	25	BP
response to hydrogen peroxide	K5	2.28E-12	23	BP
response to water deprivation	K5	2.87E-05	17	BP
response to cold	K5	0.005152	16	BP
photosynthesis, light harvesting	K5	2.15E-09	15	BP
oxidation-reduction process	B15	1.05E-07	89	BP
metabolic process	B15	0.042716	28	BP
protein folding	B15	8.73E-05	23	BP

response to stress	B15	5.58E-06	20	BP
response to oxidative stress	B15	2.53E-05	16	BP
transport	B15	0.026499	16	BP
response to high light intensity	B15	0.000477	12	BP
response to water deprivation	B15	0.001944	12	BP
response to heat	B15	0.002144	12	BP
response to hydrogen peroxide	B15	0.000212	11	BP
oxidation-reduction process	K15	0.005324	37	BP
regulation of transcription, DNA-templated	K15	3.73E-05	26	BP
isopentenyl diphosphate biosynthetic process	K15	0.000454	9	BP
defense response	K15	0.002523	9	BP
chlorophyll biosynthetic process	K15	3.32E-05	8	BP
photosynthesis	K15	0.000856	7	BP
rRNA processing	K15	6.04E-03	7	BP
pentose-phosphate shunt	K15	0.002387	7	BP
ethylene-activated signaling pathway	K15	4.35E-05	6	BP
thylakoid membrane organization	K15	0.040653	5	BP
protein folding	B30	5.2E-07	12	BP
response to stress	B30	6.32E-08	11	BP
galactolipid biosynthetic process	B30	8.89E-10	8	BP
cellular response to phosphate starvation	B30	2.11E-10	8	BP
response to heat	B30	0.000331	6	BP
phosphate ion transport	B30	4.06E-08	5	BP
negative regulation of transcription, DNA-templated	B30	0.000276	4	BP
response to hydrogen peroxide	B30	0.003228	4	BP
regulation of defense response	B30	3.07E-05	4	BP
dephosphorylation	B30	1.62E-05	4	BP
oxidation-reduction process	K30	0.035925	21	BP
carbohydrate metabolic process	K30	2.35E-05	12	BP
proteolysis	K30	0.000713	11	BP
lipid metabolic process	K30	0.000338	7	BP
defense response to bacterium	K30	0.002187	6	BP
transport	K30	0.041825	6	BP
plant-type cell wall organization	K30	0.00025	5	BP
regulation of meristem growth	K30	0.006238	4	BP
proline catabolic process	K30	1.65E-09	4	BP
glutamate biosynthetic process	K30	2.82E-07	4	BP
integral component of membrane	B2	0.00415	52	CC
cell wall	B2	2.46E-11	25	CC
apoplast	B2	5.60E-07	20	CC
plasma membrane	B2	0.035521	16	CC
cytosol	B2	0.003347	7	CC
extracellular region	B2	0.044067	7	CC
mitochondrion	B2	0.0421	6	CC

anchored component of plasma membrane	B2	0.000215	5	CC
chloroplast stroma	B2	0.005159	4	CC
anchored component of membrane	B2	0.031194	3	CC
integral component of membrane	K2	2.40E-05	105	CC
chloroplast stroma	K2	3.70E-23	76	CC
chloroplast envelope	K2	4.21E-25	67	CC
chloroplast thylakoid membrane	K2	4.48E-24	57	CC
chloroplast	K2	6.81E-05	57	CC
photosystem II	K2	1.20E-24	30	CC
apoplast	K2	8.57E-08	29	CC
photosystem I	K2	1.06E-23	28	CC
extracellular region	K2	2.11E-07	28	CC
thylakoid	K2	5.30E-16	25	CC
vacuolar proton-transporting V-type ATPase, V0 domain	B5	0.005297	2	CC
lipid particle	B5	0.007392	1	CC
integral component of membrane	K5	0.006154	110	CC
apoplast	K5	6.41E-06	29	CC
chloroplast thylakoid membrane	K5	0.000784	26	CC
extracellular region	K5	5.84E-05	26	CC
mitochondrion	K5	0.002905	23	CC
cell wall	K5	0.014704	20	CC
photosystem II	K5	2.09E-08	16	CC
plant-type cell wall	K5	0.000126	16	CC
photosystem I	K5	2.33E-07	14	CC
anchored component of plasma membrane	K5	5.40E-05	8	CC
integral component of membrane	B15	0.000281	83	CC
membrane	B15	0.036184	23	CC
cell wall	B15	3.31E-05	21	CC
apoplast	B15	0.045415	14	CC
chloroplast	B15	0.004952	14	CC
extracellular region	B15	0.005764	12	CC
mitochondrion	B15	0.003108	8	CC
Golgi apparatus	B15	0.031207	6	CC
plant-type vacuole membrane	B15	0.028245	4	CC
photosystem I reaction center	B15	0.004489	3	CC
nucleus	K15	0.013367	27	CC
chloroplast stroma	K15	1.21E-05	21	CC
chloroplast	K15	0.045595	18	CC
chloroplast envelope	K15	0.000272	15	CC
chloroplast thylakoid membrane	K15	0.000568	12	CC
extracellular region	K15	0.015725	8	CC
apoplast	K15	0.047464	7	CC
chloroplast thylakoid	K15	0.003741	5	CC
photosystem II	K15	0.001517	5	CC
plastoglobule	K15	0.003602	4	CC

cytoplasm	B30	0.000827	13	CC
cell wall	B30	0.000164	8	CC
vacuole	B30	0.002589	7	CC
apoplast	B30	0.028041	5	CC
endoplasmic reticulum lumen	B30	3.88E-07	5	CC
nucleus	B30	0.046557	4	CC
extracellular region	B30	0.020226	4	CC
plant-type cell wall	B30	0.043404	3	CC
cell periphery	B30	0.005752	2	CC
plant-type vacuole membrane	B30	0.022796	2	CC
cell wall	K30	0.00015	9	CC
apoplast	K30	0.003	7	CC
extracellular region	K30	0.002639	7	CC
vacuole	K30	0.018485	6	CC
plant-type cell wall	K30	0.00209	5	CC
anchored component of plasma membrane	K30	0.020807	2	CC
chloroplast part	K30	0.04137	2	CC
nuclear part	K30	0.000876	2	CC
nuclear matrix	K30	0.002681	2	CC
mitochondrion	K30	0.034585	2	CC
metal ion binding	B2	0.04607	29	MF
ATP binding	B2	0.000376	29	MF
transcription factor activity	B2	1.72E-05	23	MF
sequence-specific DNA binding	B2	5.84E-06	17	MF
xyloglucan:xyloglucosyl transferase activity	B2	1.22E-13	14	MF
hydrolase activity	B2	8.10E-06	13	MF
heme binding	B2	0.030486	12	MF
protein serine/threonine phosphatase activity	B2	0.000237	10	MF
2-alkenal reductase [NAD(P)] activity	B2	0.048067	9	MF
transporter activity	B2	0.043527	9	MF
ATP binding	K2	0.043347	74	MF
metal ion binding	K2	2.01E-05	66	MF
chlorophyll binding	K2	4.03E-26	32	MF
structural constituent of ribosome	K2	0.00183	30	MF
copper ion binding	K2	3.49E-06	24	MF
transporter activity	K2	0.000609	20	MF
hydrolase activity, hydrolyzing O-glycosyl compounds	K2	0.001754	14	MF
zinc ion binding	K2	8.06E-05	13	MF
peroxidase activity	K2	0.011571	11	MF
rRNA binding	K2	0.007369	10	MF
hydrolase activity	B5	0.011124	6	MF
pyridoxal phosphate binding	B5	0.001117	5	MF
transcription factor activity	B5	0.047683	5	MF
glycogen phosphorylase activity	B5	3.60E-07	4	MF

carbohydrate binding	B5	0.020675	3	MF
oxidoreductase activity, acting on paired donors	B5	0.0057	3	MF
oxidoreductase activity, acting on single donors	B5	0.000689	3	MF
protein serine/threonine phosphatase activity	B5	0.019926	3	MF
aspartyl esterase activity	B5	0.008047	2	MF
pectinesterase activity	B5	0.011467	2	MF
iron ion binding	K5	0.03487	32	MF
2-alkenal reductase [NAD(P)] activity	K5	1.35E-05	28	MF
nucleotide binding	K5	0.00621	22	MF
unfolded protein binding	K5	6.82E-08	20	MF
protein serine/threonine kinase activity	K5	0.013345	19	MF
transporter activity	K5	0.027816	18	MF
copper ion binding	K5	0.027353	17	MF
chlorophyll binding	K5	6.99E-08	16	MF
protein dimerization activity	K5	0.02655	15	MF
RNA binding	K5	0.033081	13	MF
heme binding	B15	4.92E-06	27	MF
electron carrier activity	B15	0.00852	21	MF
sequence-specific DNA binding	B15	0.003365	16	MF
copper ion binding	B15	0.003467	16	MF
peroxidase activity	B15	1.44E-08	16	MF
DNA binding	B15	0.00589	16	MF
nucleic acid binding	B15	0.045277	16	MF
ATPase activity	B15	0.000545	12	MF
RNA binding	B15	0.007776	9	MF
oxidoreductase activity, acting on paired donors	B15	0.002881	8	MF
transcription factor activity	K15	1.06E-07	23	MF
DNA binding	K15	0.003556	23	MF
ATP binding	K15	0.004963	18	MF
iron ion binding	K15	0.030247	12	MF
monooxygenase activity	K15	0.002408	10	MF
calcium ion binding	K15	0.017161	9	MF
sequence-specific DNA binding	K15	0.010919	8	MF
oxidoreductase activity, acting on paired donors	K15	0.014328	8	MF
oxidoreductase activity, acting on single donors	K15	3.98E-06	7	MF
protein binding	K15	0.00276	7	MF
ATP binding	B30	0.001651	30	MF
metal ion binding	B30	0.001165	17	MF
acid phosphatase activity	B30	9.30E-25	17	MF
protein serine/threonine kinase activity	B30	0.027207	9	MF
2-alkenal reductase [NAD(P)] activity	B30	0.000195	8	MF
unfolded protein binding	B30	0.001316	5	MF
transporter activity	B30	0.016149	5	MF
pyridoxal phosphate binding	B30	0.023044	4	MF
phosphatase activity	B30	1.65E-05	4	MF

protein serine/threonine phosphatase activity	B30	0.00794	4	MF
carbohydrate binding	K30	0.0004	6	MF
cysteine-type peptidase activity	K30	2.44E-06	6	MF
beta-galactosidase activity	K30	2.68E-07	6	MF
hydrolase activity, hydrolyzing O-glycosyl compounds	K30	0.001098	6	MF
protein binding	K30	0.004882	5	MF
copper ion binding	K30	0.026522	5	MF
monooxygenase activity	K30	0.047064	5	MF
cation binding	K30	0.001912	4	MF
xyloglucan:xyloglucosyl transferase activity	K30	0.000245	4	MF
proline dehydrogenase activity	K30	1.65E-09	4	MF

Table S4 Enriched GO terms of DEGs and comparison across stages in variety K326

See content excel file: K.allDrCK.goseq.GO.enrichment.P0.05.xlsx

Table S5 Enriched GO terms of DEGs and comparison across stages in variety BX

See content in excel file: B.allDrCK.goseq.GO.enrichment.P0.05.xlsx

Table S6 Comparison of DEGs in photosynthesis in *Nicotiana* varieties under drought

Kegg_ID	Function	DEG_id in K	DEG_id in B
K02111	F-type H+-transporting ATPase subunit alpha [EC:3.6.3.14]	K0000007g0130	--
K02638	plastocyanin	K0003030g0010	N_28075
K02639	ferredoxin	K0002373g0030<br>K0009833g0010<br>K0013140g0020	N_34854
K02692	photosystem I subunit II	K0000043g0320	--
K02694	photosystem I subunit III	K0001312g0030<br>K0005250g0030<br>K0008761g0030	--
K02695	photosystem I subunit VI	K0000351g0060<br>K0002859g0140	N_49806<br>N_66064<br>N_66878

Cellular component

r function

K02698	photosystem I subunit X	K0000100g0280<br>K0001809g0020<br>K0002991g0030	--
K02699	photosystem I subunit XI	K0007144g0010	--
K02705	photosystem II CP43 chlorophyll apoprotein	N_11965	N_32233
K02717	photosystem II oxygen-evolving enhancer protein 2	K0005601g0020	--
K02721	photosystem II PsbW protein	K0001044g0040<br>K0004899g0020	--
K03541	photosystem II 10kDa protein	K0000134g0030<br>K0001791g0030	--
K08901	photosystem II oxygen-evolving enhancer protein 3	K0004300g0170	--
K08905	photosystem I subunit V	K0001622g0160	--
K08908	light-harvesting complex I chlorophyll a/b binding protein 2	K0000073g0320	--
K08909	light-harvesting complex I chlorophyll a/b binding protein 3	K0000639g0230	--
K08910	light-harvesting complex I chlorophyll a/b binding protein 4	K0004286g0030<br>K0007189g0010	N_26609
K08912	light-harvesting complex II chlorophyll a/b binding protein 1	K0000495g0040<br>K0000592g0350<br>K0000592g0380<br>K0000982g0170<br>K0000982g0190<br>K0000982g0200<br>K0001434g0050<br>K0002210g0070<br>K0002210g0110<br>K0002229g0060<br>K0002342g0170<br>K0002814g0010<br>K0002814g0030<br>K0002814g0040<br>K0002814g0050<br>K0004269g0080	--

		K0006300g0050<br>K0023502g0010	
K08913	light-harvesting complex II chlorophyll a/b binding protein 2	K0009877g0020<br>K0029285g0010	--
K08914	light-harvesting complex II chlorophyll a/b binding protein 3	K0000297g0150<br>K0000639g0060<br>K0008360g0020<br>K0012832g0010	N_11654
K08915	light-harvesting complex II chlorophyll a/b binding protein 4	K0011597g0020	--
K08917	light-harvesting complex II chlorophyll a/b binding protein 6	K0002865g0040<br>K0003434g0040	--
K14332	photosystem I subunit PsaO	K0000937g0110<br>K0003068g0060	N_15478<br>N_51558
Total	23	56	10

Table S7 Comparison of the DEGs encoding proteins in hormone signal transduction in response to drought stress in *Nicotiana* varieties

Kegg_ID	Function	DEGs in K326	DEGs in BX	Hormone pathway
K14432	ABA responsive element binding factor (ABF)	K0000287g0240<br>K0002760g0050<br>K0002915g0050	N_20477<br>N_34019<br>N_45528<br>N_53972	Abscisic acid

			N_55157<br>N_60579	
K14496	abscisic acid receptor PYR/PYL family	K0000256g0380<br>K0001098g0070<br>K0002903g0010<br>K0003710g0010<br>K0005335g0030<br>K0007288g0010<br>K0026691g0010	--	Abscisic acid
K14497	protein phosphatase 2C [EC:3.1.3.16] (PP2C)	K0000153g0190<br>K0000463g0070<br>K0000647g0160<br>K0000714g0020<br>K0001231g0090<br>K0001519g0160<br>K0005632g0010<br>K0005987g0010<br>K0008967g0020<br>K0009622g0010<br>K0013277g0010	N_12337<br>N_15421<br>N_17495<br>N_32381<br>N_33098<br>N_33100<br>N_33992<br>N_48823<br>N_56959<br>N_58008<br>N_65819<br>N_67771<br>N_68756<br>N_7726<br>N_7732	Abscisic acid
K14498	serine/threonine-protein kinase SRK2 [EC:2.7.11.1] (SnRk2)	K0002604g0060	N_14073<br>N_49501<br>N_8876	Abscisic acid
K13946	auxin influx carrier (AUX1 LAX family)	K0009993g0010	N_40954<br>N_66808	Auxin
K14484	auxin-responsive protein IAA (IAA)	K0002327g0080<br>K0002424g0010<br>K0003159g0030<br>K0018322g0010	N_10115<br>N_12426<br>N_41117<br>N_51161<br>N_51162<br>N_63867	Auxin
K14486	auxin response factor (ARF)	--	N_28379	Auxin
K14487	auxin responsive GH3 gene family (GH3)	K0002370g0050	N_34041	Auxin
K14488	SAUR family protein	K0000980g0430<br>K0002096g0080<br>K0006348g0020<br>K0011271g0080	N_70191	Auxin
K14503	brassinosteroid resistant 1/2 (BZR1/2)	K0000628g0120	N_12440	Brassinosteroid
K14505	cyclin D3, plant (CYCD3)	K0004339g0110	--	Brassinosteroid

K14489	Arabidopsis histidine kinase 2/3/4 (cytokinin receptor) [EC:2.7.13.3] (CRE)	K0002951g0010<br>K0003884g0020<br>N_5095	--	Cytokinine
K14490	histidine-containing phosphotransferase (AHP)	K0008107g0010	N_21254<br>N_50130	Cytokinine
K14492	two-component response regulator ARR-A family (A-ARR)	K0000571g0150<br>K0000688g0210<br>K0002162g0080	N_38908<br>N_52852	Cytokinine
K14516	ethylene-responsive transcription factor 1 (ERF)	K0000044g0100	N_22140	Ethylene
K14493	gibberellin receptor GID1	K0002093g0090<br>K0003058g0050	N_15733	Gibberellin
K14494	DELLA protein	K0007567g0050	--	Gibberellin
K16189	phytochrome-interacting factor 4	--	N_62241<br>N_62244	Gibberellin
K13464	jasmonate ZIM domain-containing protein (JAZ)	K0000238g0080<br>K0001546g0010<br>N_16446	N_11068<br>N_6183<br>N_9177	Jasmonic acid
K14506	jasmonic acid-amino synthetase (JAR)	--	N_26602<br>N_71459<br>N_71465	Jasmonic acid
K13422	transcription factor MYC2	K0000804g0160	--	Jasmonic acid
K13449	pathogenesis-related protein 1 (PR1)	K0005400g0020	--	Salicylic acid
K14431	transcription factor TGA (TGA)	K0004756g0060	--	Salicylic acid
Total	24	51	50	8

Table S8 DEGs and involved pathway node in the correlation network

Gene.EncodedProtein_in_K326	Gene2.EncodedProtein_in BX	Pathway node
K0000153g0190.PP2C	N_12337.PP2C	Abscisic acid pathway
K0000256g0380.PYR	N_14073.SnRk2	Abscisic acid pathway
K0000287g0240.ABF	N_15421.PP2C	Abscisic acid pathway
K0000463g0070.PP2C	N_17495.PP2C	Abscisic acid pathway
K0000647g0160.PP2C	N_20477.ABF	Abscisic acid pathway
K0000714g0020.PP2C	N_32381.PP2C	Abscisic acid pathway
K0001098g0070.PYR	N_33098.PP2C	Abscisic acid pathway
K0001231g0090.PP2C	N_33100.PP2C	Abscisic acid pathway
K0001519g0160.PP2C	N_33992.PP2C	Abscisic acid pathway
K0002604g0060.SnRk2	N_34019.ABF	Abscisic acid pathway
K0002760g0050.ABF	N_45528.ABF	Abscisic acid pathway
K0002903g0010.PYR	N_48823.PP2C	Abscisic acid pathway
K0002915g0050.ABF	N_49501.SnRk2	Abscisic acid pathway

K0003710g0010.PYR	N_53972.ABF	Abscisic acid pathway
K0005335g0030.PYR	N_55157.ABF	Abscisic acid pathway
K0005632g0010.PP2C	N_56959.PP2C	Abscisic acid pathway
K0005987g0010.PP2C	N_58008.PP2C	Abscisic acid pathway
K0007288g0010.PYR	N_60579.ABF	Abscisic acid pathway
K0008967g0020.PP2C	N_65819.PP2C	Abscisic acid pathway
K0009622g0010.PP2C	N_67771.PP2C	Abscisic acid pathway
K0013277g0010.PP2C	N_68756.PP2C	Abscisic acid pathway
K0026691g0010.PYR	N_7726.PP2C	Abscisic acid pathway
	N_7732.PP2C	Abscisic acid pathway
	N_8876.SnRk2	Abscisic acid pathway
K0000980g0430.SAUR	N_10115.IAA	Auxin pathway
K0002096g0080.SAUR	N_12426.IAA	Auxin pathway
K0002327g0080.IAA	N_28379.ARF	Auxin pathway
K0002370g0050.GH3	N_34041.GH3	Auxin pathway
K0002424g0010.IAA	N_40954.AUX	Auxin pathway
K0003159g0030.IAA	N_41117.IAA	Auxin pathway
K0006348g0020.SAUR	N_51161.IAA	Auxin pathway
K0009993g0010.AUX	N_51162.IAA	Auxin pathway
K0011271g0080.SAUR	N_63867.IAA	Auxin pathway
K0018322g0010.IAA	N_66808.AUX	Auxin pathway
	N_70191.SAUR	Auxin pathway
K0000628g0120.BZR	N_12440.BZR	Brassinosteroid pathway
K0004339g0110.CYCD		Brassinosteroid pathway
K0000571g0150.A-ARR	N_21254.AHP	Cytokinine pathway
K0000688g0210.A-ARR	N_38908.A-ARR	Cytokinine pathway
K0002162g0080.A-ARR	N_50130.AHP	Cytokinine pathway
K0002951g0010.CRE	N_52852.A-ARR	Cytokinine pathway
K0003884g0020.CRE		Cytokinine pathway
N_5095.CRE		Cytokinine pathway
K0002093g0090.GID	N_15733.GID	Gibberellin pathway
K0003058g0050.GID	N_62241.TF	Gibberellin pathway
K0007567g0050.DELLA	N_62244.TF	Gibberellin pathway
K0008107g0010.GID		Gibberellin pathway

Supplementary figures

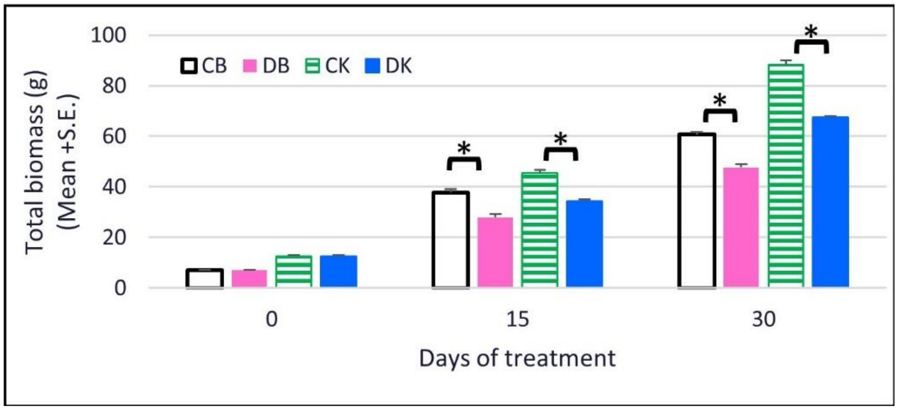

Figure S1. Biomass changes of whole plant of two *N.* varieties under drought

CK, DK, CB and DB represent the control K326, drought treated K326, control Basma, drought treated Basma. The number followed the CK, DK, CB, DB represent the treating stages. * for statistical significance at $p < 0.05$. S.E. for standard error.

A

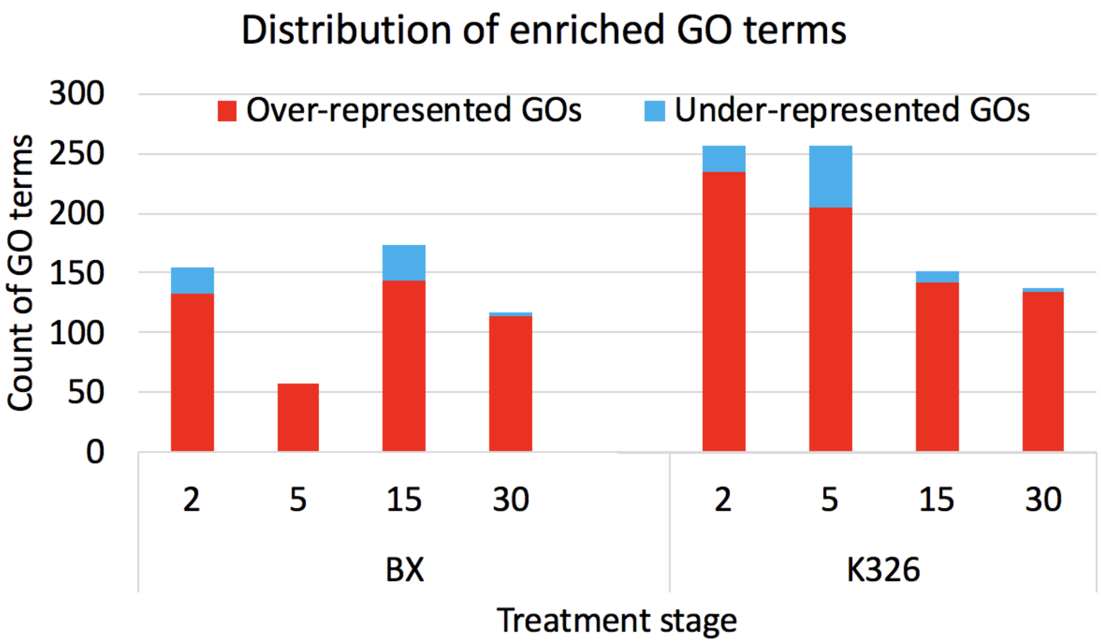

B

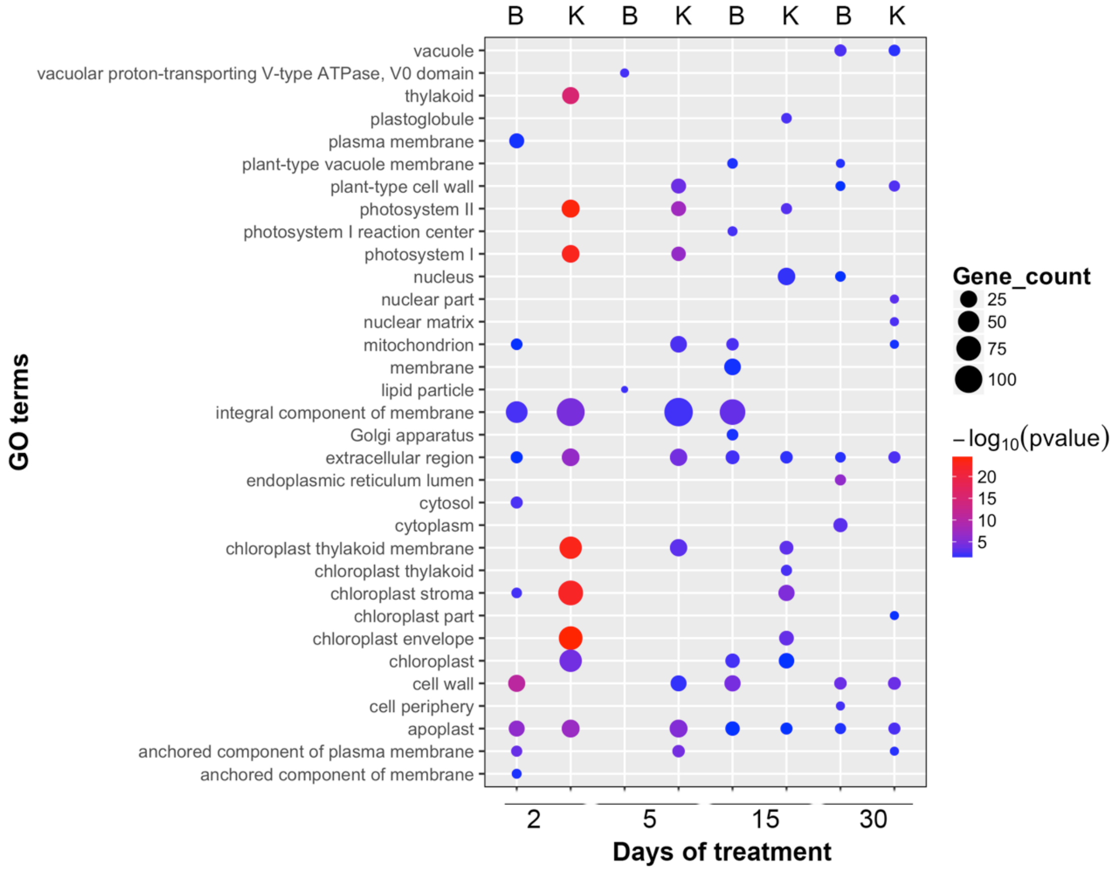
B K B K B K B K
vacuole
vacuolar proton-transporting V-type ATPase, V0 domain
thylakoid
plastoglobule
plasma membrane
plant-type vacuole membrane
plant-type cell wall
photosystem II
photosystem I reaction center
photosystem I
nucleus
nuclear part
nuclear matrix
mitochondrion
membrane
lipid particle
integral component of membrane
Golgi apparatus
extracellular region
endoplasmic reticulum lumen
cytosol
cytoplasm
chloroplast thylakoid membrane
chloroplast thylakoid
chloroplast stroma
chloroplast part
chloroplast envelope
chloroplast
cell wall
cell periphery
apoplast
anchored component of plasma membrane
anchored component of membrane
GO terms
2
5
15
30
Days of treatment
Gene_count
25
50
75
100
$-\log_{10}$(pvalue)
20
15
10
5

C

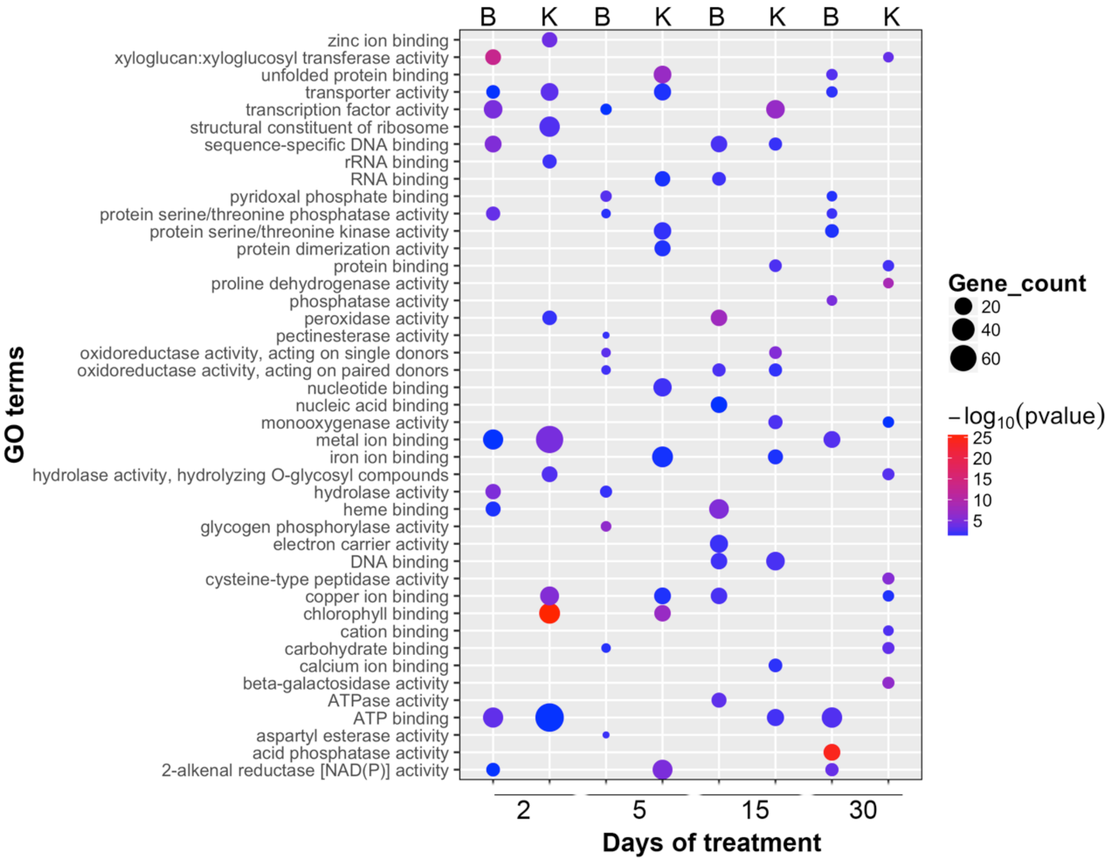
B K B K B K B K
zinc ion binding
xyloglucan:xyloglucosyl transferase activity
unfolded protein binding
transporter activity
transcription factor activity
structural constituent of ribosome
sequence-specific DNA binding
rRNA binding
RNA binding
pyridoxal phosphate binding
protein serine/threonine phosphatase activity
protein serine/threonine kinase activity
protein dimerization activity
protein binding
proline dehydrogenase activity
phosphatase activity
peroxidase activity
pectinesterase activity
oxidoreductase activity, acting on single donors
oxidoreductase activity, acting on paired donors
nucleotide binding
nucleic acid binding
monooxygenase activity
metal ion binding
iron ion binding
hydrolase activity, hydrolyzing O-glycosyl compounds
hydrolase activity
heme binding
glycogen phosphorylase activity
electron carrier activity
DNA binding
cysteine-type peptidase activity
copper ion binding
chlorophyll binding
cation binding
carbohydrate binding
calcium ion binding
beta-galactosidase activity
ATPase activity
ATP binding
aspartyl esterase activity
acid phosphatase activity
2-alkenal reductase [NAD(P)] activity
GO terms
2
5
15
30
Days of treatment
Gene_count
20
40
60
$-\log_{10}(\text{pvalue})$
25
20
15
10
5

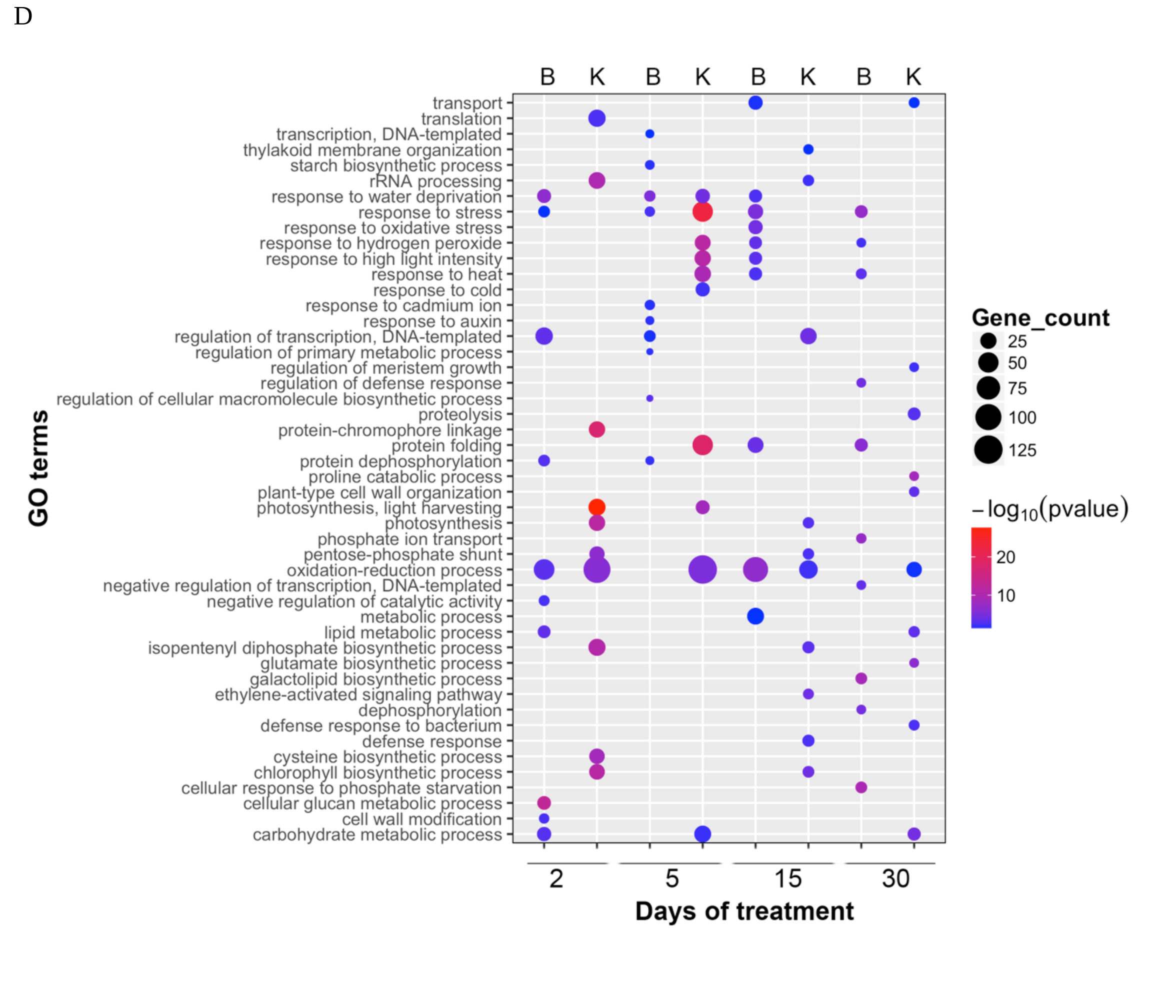

Figure S2. Comparison of top enriched GO terms of DEGs in response to drought

(**A**) More enriched GO terms ($p < 0.05$) in variety K326 than Basma (BX). (**B**), (**C**) and (**D**) The GOs comparison in category of cellular component, molecular function and biological process, respectively. The B and K in the top of GO comparison represent variety BX and K326, respectively. Only the top ten enriched GOs at each treatment stages were plotted.

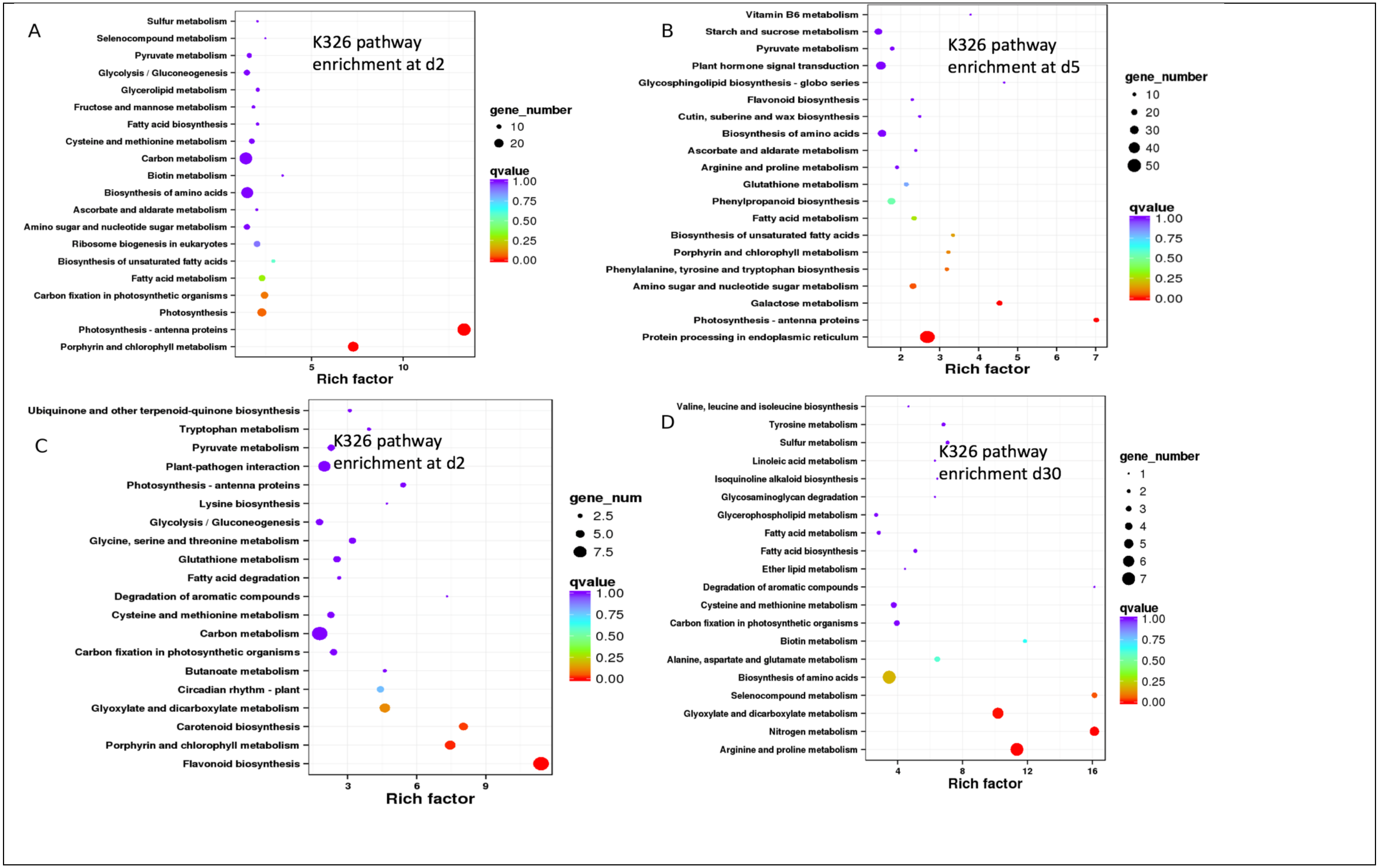
A
K326 pathway enrichment at d2
Sulfur metabolism
Selenocompound metabolism
Pyruvate metabolism
Glycolysis / Gluconeogenesis
Glycerolipid metabolism
Fructose and mannose metabolism
Fatty acid biosynthesis
Cysteine and methionine metabolism
Carbon metabolism
Biotin metabolism
Biosynthesis of amino acids
Ascorbate and aldarate metabolism
Amino sugar and nucleotide sugar metabolism
Ribosome biogenesis in eukaryotes
Biosynthesis of unsaturated fatty acids
Fatty acid metabolism
Carbon fixation in photosynthetic organisms
Photosynthesis
Photosynthesis - antenna proteins
Porphyrin and chlorophyll metabolism
5
10
Rich factor
gene_number
10
20
qvalue
1.00
0.75
0.50
0.25
0.00
B
K326 pathway enrichment at d5
Vitamin B6 metabolism
Starch and sucrose metabolism
Pyruvate metabolism
Plant hormone signal transduction
Glycosphingolipid biosynthesis - globo series
Flavonoid biosynthesis
Cutin, suberine and wax biosynthesis
Biosynthesis of amino acids
Ascorbate and aldarate metabolism
Arginine and proline metabolism
Glutathione metabolism
Phenylpropanoid biosynthesis
Fatty acid metabolism
Biosynthesis of unsaturated fatty acids
Porphyrin and chlorophyll metabolism
Phenylalanine, tyrosine and tryptophan biosynthesis
Amino sugar and nucleotide sugar metabolism
Galactose metabolism
Photosynthesis - antenna proteins
Protein processing in endoplasmic reticulum
2 3 4 5 6 7
Rich factor
gene_number
10
20
30
40
50
qvalue
1.00
0.75
0.50
0.25
0.00
C
K326 pathway enrichment at d2
Ubiquinone and other terpenoid-quinone biosynthesis
Tryptophan metabolism
Pyruvate metabolism
Plant-pathogen interaction
Photosynthesis - antenna proteins
Lysine biosynthesis
Glycolysis / Gluconeogenesis
Glycine, serine and threonine metabolism
Glutathione metabolism
Fatty acid degradation
Degradation of aromatic compounds
Cysteine and methionine metabolism
Carbon metabolism
Carbon fixation in photosynthetic organisms
Butanoate metabolism
Circadian rhythm - plant
Glyoxylate and dicarboxylate metabolism
Carotenoid biosynthesis
Porphyrin and chlorophyll metabolism
Flavonoid biosynthesis
3 6 9
Rich factor
gene_num
2.5
5.0
7.5
qvalue
1.00
0.75
0.50
0.25
0.00
D
K326 pathway enrichment d30
Valine, leucine and isoleucine biosynthesis
Tyrosine metabolism
Sulfur metabolism
Linoleic acid metabolism
Isoquinoline alkaloid biosynthesis
Glycosaminoglycan degradation
Glycerophospholipid metabolism
Fatty acid metabolism
Fatty acid biosynthesis
Ether lipid metabolism
Degradation of aromatic compounds
Cysteine and methionine metabolism
Carbon fixation in photosynthetic organisms
Biotin metabolism
Alanine, aspartate and glutamate metabolism
Biosynthesis of amino acids
Selenocompound metabolism
Glyoxylate and dicarboxylate metabolism
Nitrogen metabolism
Arginine and proline metabolism
4 8 12 16
Rich factor
gene_number
1
2
3
4
5
6
7
qvalue
1.00
0.75
0.50
0.25
0.00

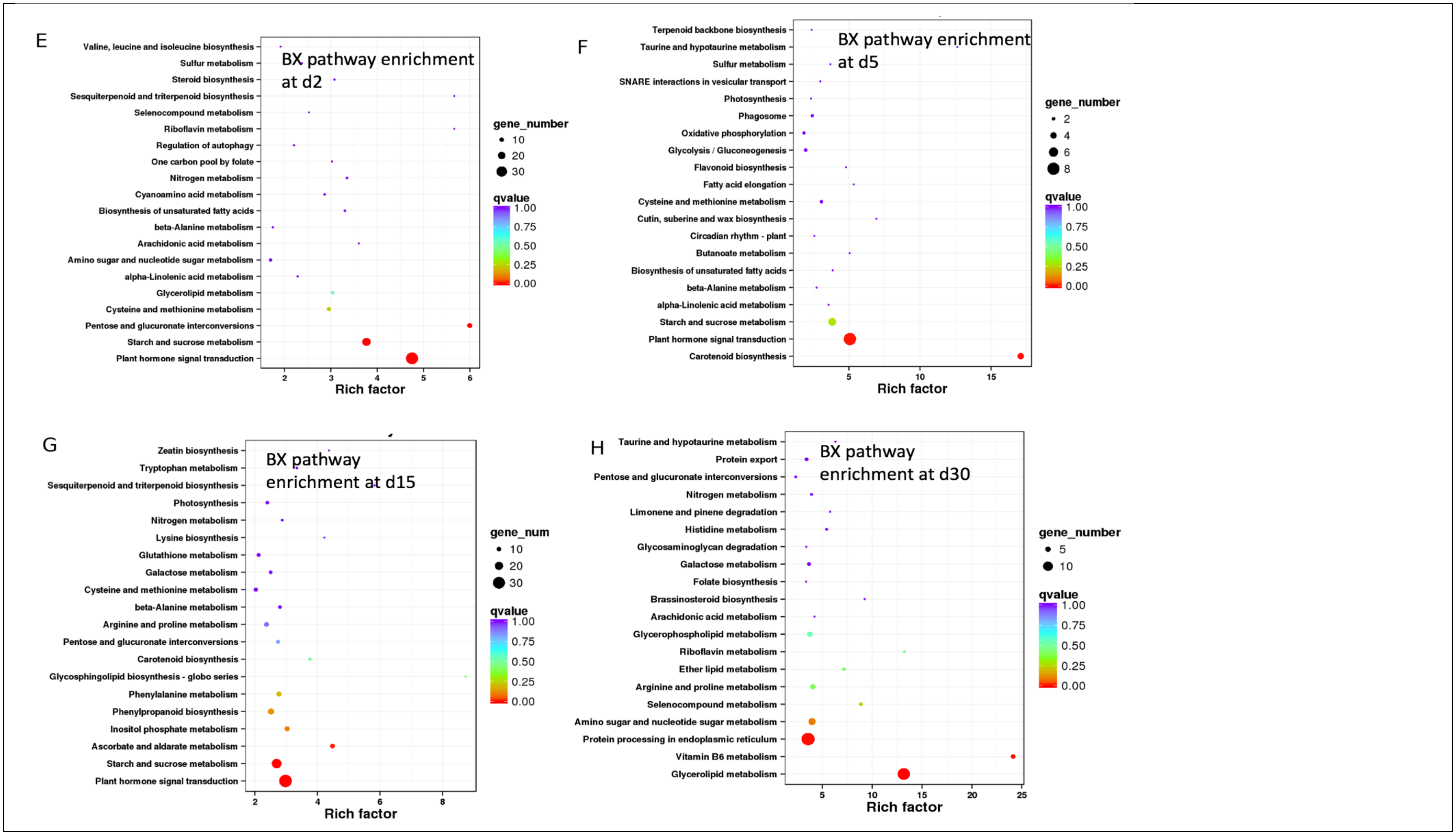

Figure S3. Comparison of enriched metabolism pathways involved by DEGs under drought

The significant enrichment ($p<0.05$) was shown only. K2, K5, K15, and K30 represent the stages after 2, 5, 15 and 30 days of drought treatment in variety K326. B2, B5, B15, and B30 represent the stages after 2, 5, 15 and 30 days of drought treatment in variety BX. The Rich factor is the percent ratio of count of differentially expressed genes to total count of genes in a pathway. The greater rich factor represents a greater enrichment.

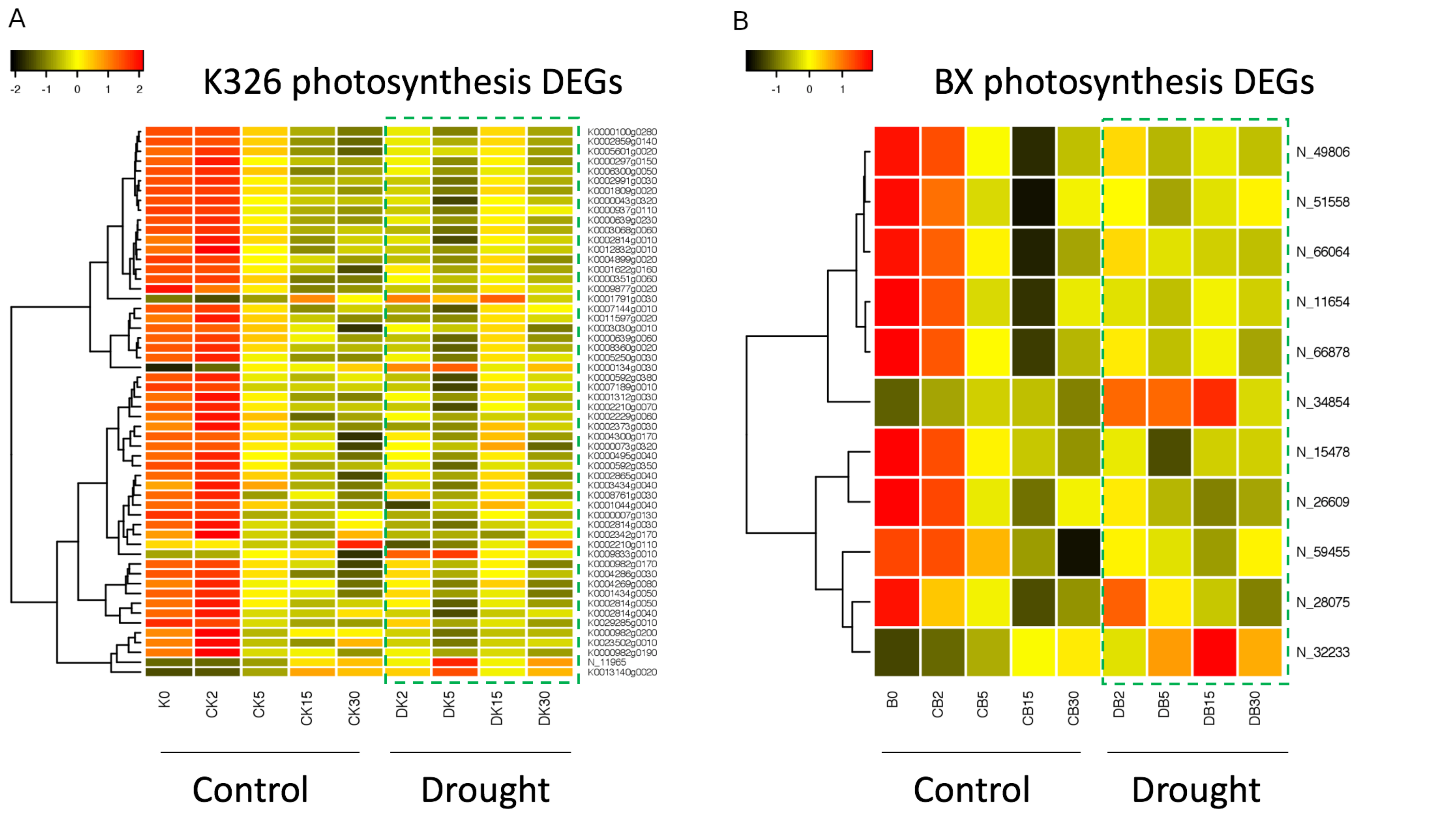

Figure S4 Comparison of expression patterns of DEGs in photosynthesis under drought

The heatmap shows Z-score of each differentially expressed gene after log_2(FPKM+1) transformation of the mean FPKM value of three experimental replicates. The ID of each gene was shown in the right of each row and each column represents an experimental condition. 0, 2, 5, 15, and 30 represent the days of treatment. K326 and BX represents for variety K326 and Basma. (**A**) expression patterns of DEGs in K326; (**B**) expression patterns of DEGs in K326.

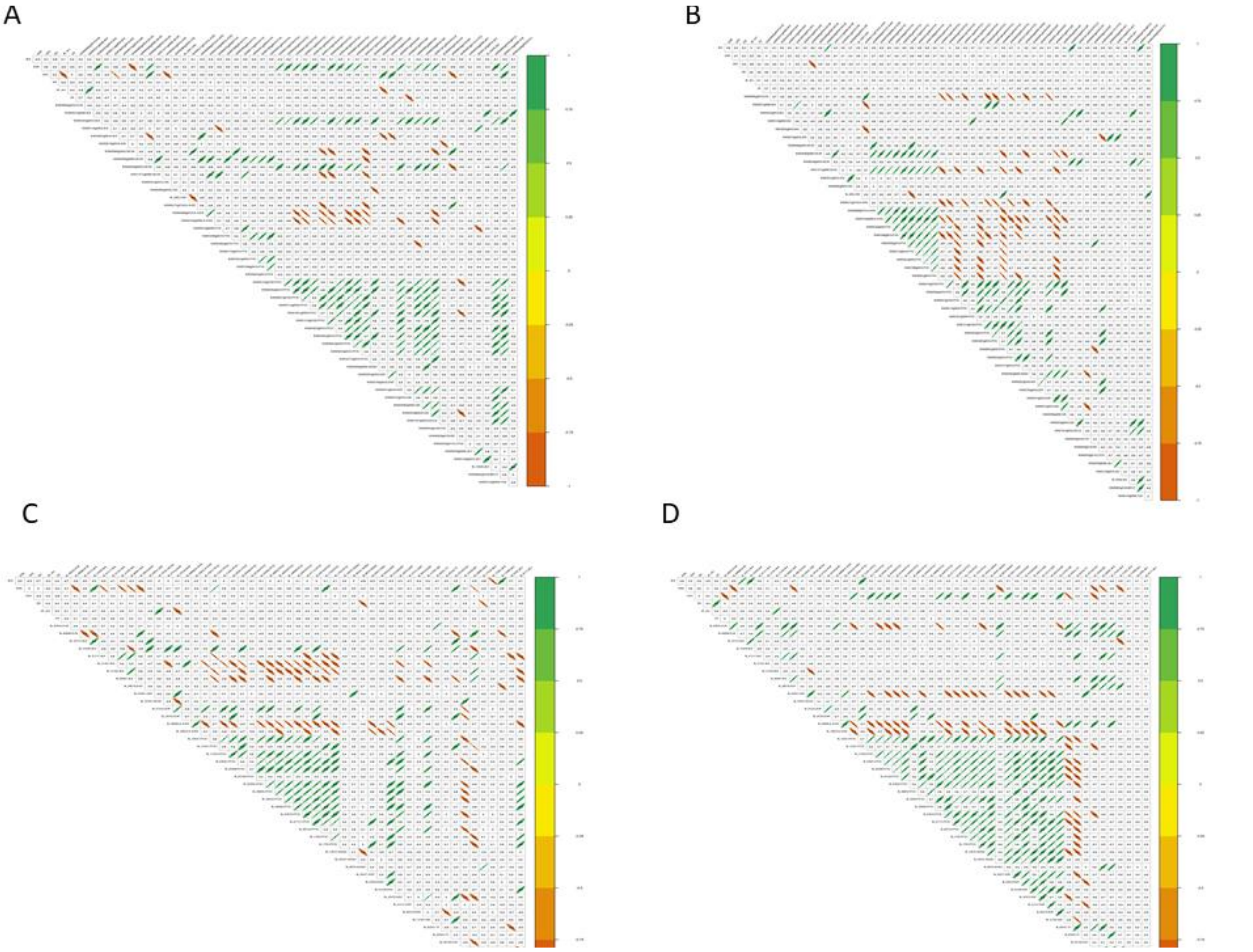
A
B
C
D

Figure S5 Correlation network of hormones and DEGs in hormones signaling pathways

(**a**) The correlation network showing crosstalk of differentially expressed genes between hormone signaling pathways in variety K326 under control ; (**b**) The correlation network showing crosstalk of differentially expressed genes between hormone signaling pathways in variety K326 under drought stress ; (**c**) The correlation network showing crosstalk of differentially expressed genes between hormone signaling pathways in variety BX under control ; (**d**) The correlation network showing crosstalk of differentially expressed genes between hormone signaling pathways in variety BX under drought stress.
